# *Sucrose non-fermenting related kinase* enzyme is essential for cardiac metabolism

**DOI:** 10.1242/bio.20149811

**Published:** 2014-12-12

**Authors:** Stephanie M. Cossette, Adam J. Gastonguay, Xiaoping Bao, Alexandra Lerch-Gaggl, Ling Zhong, Leanne M. Harmann, Christopher Koceja, Robert Q. Miao, Padmanabhan Vakeel, Changzoon Chun, Keguo Li, Jamie Foeckler, Michelle Bordas, Hartmut Weiler, Jennifer Strande, Sean P. Palecek, Ramani Ramchandran

**Affiliations:** 1 Department of Pediatrics, Developmental Vascular Biology Program, Children’s Research Institute, Medical College of Wisconsin, Milwaukee, WI 53226, USA; 2 Department of Chemical and Biological Engineering, University of Wisconsin, Madison, WI 53706, USA; 3 Division of Pediatric Pathology, Medical College of Wisconsin, Milwaukee, WI 53226, USA. Department of Pathology, Medical College of Wisconsin, Milwaukee, WI 53226, USA; 4 Division of Cardiovascular Medicine, Cardiovascular Center, Medical College of Wisconsin, Milwaukee, WI 53226, USA. Clinical and Translational Science Institute, Medical College of Wisconsin, Milwaukee, WI 53226, USA; 5 Division of Pediatric Surgery, Medical College of Wisconsin, Milwaukee, WI 53226, USA. Department of Surgery, Medical College of Wisconsin, Milwaukee, WI 53226, USA; 6 Division of Pediatric Pathology, Medical College of Wisconsin, Milwaukee, WI 53226, USA. Department of Pathology, Medical College of Wisconsin, Milwaukee, WI 53226, USA; 7 Division of Nephrology, Hypertension and Renal Transplantation, College of Medicine, University of Florida, Gainesville, FL 32610, USA. Department of Medicine, College of Medicine, University of Florida, Gainesville, FL 32610, USA; 8 Blood Research Institute, BloodCenter of Wisconsin, Milwaukee, WI 53226, USA; 9 Division of Cardiovascular Medicine, Cardiovascular Center, Medical College of Wisconsin, Milwaukee, WI 53226, USA. Department of Cell Biology, Neurobiology and Anatomy, Medical College of Wisconsin, Milwaukee, WI 53226, USA; 10 Department of Obstetrics and Gynecology, Medical College of Wisconsin, Milwaukee, WI 53226, USA

**Keywords:** Cardiac, Metabolism, SNRK, Fatty acid oxidation, tie2

## Abstract

In this study, we have identified a novel member of the AMPK family, namely *Sucrose non-fermenting related kinase* (*Snrk*), that is responsible for maintaining cardiac metabolism in mammals. SNRK is expressed in the heart, and brain, and in cell types such as endothelial cells, smooth muscle cells and cardiomyocytes (CMs). *Snrk* knockout (KO) mice display enlarged hearts, and die at postnatal day 0. Microarray analysis of embryonic day 17.5 *Snrk* hearts, and blood profile of neonates display defect in lipid metabolic pathways. *SNRK* knockdown CMs showed altered phospho-acetyl-coA carboxylase and phospho-AMPK levels similar to global and endothelial conditional KO mouse. Finally, adult cardiac conditional KO mouse displays severe cardiac functional defects and lethality. Our results suggest that *Snrk* is essential for maintaining cardiac metabolic homeostasis, and shows an autonomous role for SNRK during mammalian development.

## INTRODUCTION

Highly metabolic tissues require adequate oxygenation for aerobic metabolism. Therefore, it is not surprising that angiogenesis, the process of neo-vessel formation, and metabolism, process within a living cell that is necessary for maintenance of cellular physiology, is intertwined at the cellular and molecular level. Blood vessels of the circulatory system are the primary route of metabolite distribution, and cardiomyocyte (CM or CMs) of the heart are the primary recipient cell of the metabolites for maintaining energy requirements. Endothelial cells thus are hypothesized to communicate with CMs to maintain metabolic homeostasis in the heart (Wang et al., 2013; Yagyu et al., 2003). Interestingly, CM metabolism is developmentally dynamic in that the fetal heart primarily utilizes glucose as its major energy substrate, and during postnatal maturation, the heart switches to primarily utilizing fatty acid oxidation (FAO) to meet its energy demands (Breckenridge et al., 2013; Lopaschuk and Jaswal, 2010). However, the underlying mechanisms contributing to the energy source switch in mammals is not known. Perhaps, proteins that participate in both metabolic and angiogenesis pathways are candidate for this mechanism. The peroxisome proliferator-activated receptors (PPARs), and their co-activator PPAR-gamma co-activator 1-alpha (PGC-1α) have been reported as a master metabolic sensor that participates in angiogenesis in vivo (Arany et al., 2008; Garrido-Urbani et al., 2011). Similarly, two metabolic sensors, AMP-activated protein kinase (AMPK) (Jäger et al., 2007) and Silent mating type information regulation 1 (SIRT1) (Rodgers et al., 2005) also participate in metabolism and angiogenesis pathways, by directly regulating PGC-1α activity. Although master metabolic sensors have been identified, the mechanisms that control the switch in cardiac energy state from embryonic tissue to adult during cardiovascular development are not known. Here, we provide evidence that a novel member of the AMPK family namely sucrose non-fermenting related kinase (SNRK), previously identified as essential for angiogenesis (Chun et al., 2009) is likely involved in this critical step, and maintains metabolic homeostasis via regulation of the phosphorylated acetyl-coA carboxylase (pACC)-phosphorylated AMPK (pAMPK) pathway during this transitional period in development.

Human (Labastie et al., 1998) and mouse (Kertesz et al., 2002) *SNRK* is expressed in developing endothelial cells in the embryonic yolk sac, and in embryonic coronary endothelial, smooth muscle and CMs. SNRK is a substrate for Liver kinase B1 (LKB1) via phosphorylation at threonine residue 173 (Jaleel et al., 2005), and has recently been implicated as an inhibitor of colon cancer cell proliferation (Rines et al., 2012) as well as adipocyte inflammation (Li et al., 2013). To date, there is no report of SNRK function in mammalian development. Here, we report the generation of a global and conditional *Snrk* knockout (KO) mouse. Extensive characterization of defects at embryonic day (E) 17.5 and postnatal day 0 (P0) stages has been performed. The global KO mice die at P0, show enlarged hearts and lethality is associated with metabolic defects in cardiac tissues. Furthermore, adult cardiac specific conditional *Snrk* KO mice display severe cardiac functional deficits and lethality. Mechanistically, the pACC-pAMPK pathway is deregulated in *SNRK* knockdown CMs in vitro, and in *Snrk* KO and endothelial conditional *Snrk* KO hearts in vivo. Collectively, these results suggest that SNRK function is essential in endothelial cells, and triggers changes in metabolic pathways that affect cardiac function later in adult. Thus, SNRK is a critical regulator of cardiac energy homeostasis during cardiovascular development.

## MATERIALS AND METHODS

### Mouse experiments

The mice were housed in the Medical College of Wisconsin Biological Resource Center, and all experiments were performed in accordance with an Institutional Animal Care and Use Committee approved animal procedure protocol 1022. For the global loss of *Snrk* experiments, embryos were isolated from *Snrk* heterozygous (HET) mice mating, and were staged according to the presence of vaginal plug (stage E0.5). Embryos were collected at E10.5, E12.5, E15.5 and E17.5, and mouse neonate pups were collected at P0, P1 and P3 for genotype and phenotype analysis. For the conditional specific loss of *Snrk* experiments, neonates were collected from either MYH6CRE or TIE2CRE positive *Snrk* LoxP/WT males mated to *Snrk* LoxP/LoxP females. Litter matched embryos/mice were used for each animal experiment. Mixed backcrossed animals were used for the global *Snrk* KO experiments and non-backcrossed animals were used for all of the conditional null experiments.

### *Snrk* KO mouse generation

The mouse genomic locus for *Snrk* was isolated from BAC22R1 (Roswell Park Institute). PCR reactions including specific restriction sites were used to clone a 7478 bp DNA fragment containing *Snrk* genomic sequence encompassing 1880 bp upstream and 5007 bp downstream of exon 3 into pL251 plasmid. A mini-targeting vector containing *Snrk* homologous sequence flanking a neomycin (Neo) resistance cassette was used to replace Exon 3. This new plasmid called *Snrk* KO was linearized and transfected into mouse embryonic stem cells (mESCs). Transfected cells were then subjected to selection using neomycin resistance (G418; EMD Millipore). mESCs were screened for successful targeting and integration using PCR with the following primers, *Snrk* WT 5 prime end forward 5′-GTGACAGAATGGTCTTCAGGAACC-3′; *Snrk* KO 5 prime end reverse 5′-GGAAGGTGCCACTCCCACTG-3′; *Snrk* KO 3 prime end forward 5′-GACAGGTCGGTCTTGACAAAAAG-3′, and *Snrk* WT 3 prime end reverse 5′-TAACAGCAGCAGATGCCACCAG-3′. Chimeric mice were generated using the *Snrk* KO positive mESCs. *Snrk* KO positive chimeric mice were backcrossed with C57BL/6 (JackMice 000664) to generate germline transmissible *Snrk* KO heterozygous mice.

### *Snrk* conditional (cKO) mouse generation

The mouse genomic locus for *Snrk* was isolated from BAC22R1 (Roswell Park Institute). PCR reactions including specific restriction sites were used to clone a 7478 bp DNA fragment containing *Snrk* genomic sequence encompassing 1880 bp upstream and 5007 bp downstream of Exon 3 into pL251 plasmid. To create the conditional construct, LoxP sites were introduced into the genomic sequence flanking Exon 3 and a neomycin (Neo) cassette with flanking flippase recombination sites (FRT) were inserted downstream of Exon 3. This mini-targeting vector containing *Snrk* homologous sequence flanking the conditional construct was used to replace Exon 3. This new plasmid called *Snrk* LoxP was linearized and transfected into mouse embryonic stem cells (mESCs). Transfected cells were then subjected to selection using neomycin resistance (G418; EMD Millipore). mESCs were screened for successful targeting and integration using PCR with the following primers, *Snrk* WT 5 prime end forward 5′-GTGACAGAATGGTCTTCAGGAACC-3′; *Snrk* Neo 5 prime end reverse 5′-GGAAGGTGCCACTCCCACTG-3′; *Snrk* Neo 3 prime end forward 5′-GGACAGGTCGGTCTTGACAAAAG-3′, and *Snrk* WT 3 prime end reverse 5′-TAACAGCAGCAGATGCCACCAG-3′. Chimeric mice were generated using the *Snrk* LoxP positive ESCs. *Snrk* LoxP positive chimeric mice were backcrossed with C57BL/6 (JackMice 000664) to generate germline transmissible *Snrk* LoxP heterozygous mice.

### Genotyping PCR primers

*Snrk* KO primers: WT-Forward 5′-CATTGTGCTGAGTGGCTTGGAG-3′, WT-Reverse 5′-TCCTCGCTTGAACCCTGCCA-3′, KO-Forward 5′-ATGGCTTCTGAGGCGGAAAG-3′ (WT 713 bp, KO 528 bp). *Snrk* LoxP Primers: *Snrk* WT forward 5′-AAACTAGTGCAGCACCCCAA-3′, *Snrk* WT reverse 5′-TGAAATAACCTGTCTGCCAGC-3′, *Snrk* LoxP reverse 5′-AGGAACTTCATCAGTCAGGTACA-3′ (WT 591 bp, LoxP 551 bp). TIE2CRE primers: Forward 5′-CGCATAACCAGTGAAACAGCATTGC-3′, Reverse 5′-CCCTGTGCTCAGACAGAAATGAGA-3′ (450 bp). MYH6CRE primers: Forward 5′-ATGACAGACAGATCCCTCCTATCTCC-3′, Reverse 5′-CTCATCACTCGTTGCATCATCGAC-3′ (300 bp). HPRT control primers: Forward 5′-AGCGCAAGTTGAATCTGC-3′, Reverse 5′-AGCGACAATCTACCAGAG-3′ (219 bp). *Snrk* LoxP Excision Primers: Forward 5′-TGCTGGGGTCTCTCACCATA-3′, Reverse 5′-TGGGGCAAAGTTCCCATCTG-3′ (Unexcised 1542 bp, Excised 555 bp).

### Southern blot

Genomic DNA was isolated from E10.5, E12.5 and E15.5 *Snrk* WT, HET and KO embryos. The 5 µg of DNA was digested for 6 hours with the restriction enzyme BamHI. DNA fragments were separated on a 0.7% agarose gel. The DNA fragments were transfer, UV-linked to a Zeta-probe GT membrane (BIO-RAD) followed by Southern blot hybridization using a 669 bp labeled probe. The sequence for the Southern blot probe is provided below. 5′-AGTAACCCTGACCATCCCTTCCTGCAAATGTCCTTTAACAATCTTGTCACACAACATTGGGGTGGCAACATGGCTATTAGAAATTGGACTCATGCTAATTGTGACCTTGACTAAGTAACTGACCACTCTGCTGTTTCTTTGTCTGTAAGATGGACAGCTTTCCTATGGTATTTTGTTTGCTTTTTCATTGTACAGATGGGAACTTTGCCCCATCACCATGGGAACAAAGGGCAGACTAGGAAACTGCTGTGCTACATGCCAGTGTATGTGGGGCTCTTGGTATGGGGCCCTTAGCATTCCCAGCTGTCCTTGCCTTCTCCCCTAGCATCCTTCGTTCTCTTGTCGACAGTTACCTGTCTGTAGTGGAAGGACTTGCTGTTTTTCAGGTTAGATTAGCTTTGTTCCCTTAAACCATATCAGTTGTGAAGGTCATACAAGAAATTGTTCAGACTTGAAAGGATTTGTTGAACTGAAGGTGCCATCAGCTTTAGATTTTATTTAATCTCAGAGAAACATATTCTTAGGTCACATGAACAGGACATCAGACTTCATAGACAAGAGTGTTGATATGTGCGCTGCAGGCCACAGGAGATGAGTGAGAACAACATGCTGACATGTTAGACTGACGATGTCACATAAAACATGGTGGAGGGAGGCAGAATTTGTTAG-3′.

### RT-PCR

RNA was collected from E12.5 mouse embryos from HET (n = 3), WT (n = 3) and KO (n = 3) and E17.5 heart tissues from WT (n = 3), KO (n = 3) and HET (n = 3) embryos using TRIzol reagent (LifeTechnologies). NanoDrop ND-2000 was used to determine RNA quantity and quality. For the semi-quantitative PCR *Snrk* exons 3–7 and *glyceraldehyde 3-phosphate dehydrogenase* (*Gapdh*). The PCR primers used: *Snrk* exon 3 forward 5′-ACCACGAGCTGCGGGTCTCT-3′, *Snrk* exon 7 reverse 5′-GACACTTGCGCCCACTGGCT-3′, *Gapdh* forward 5′-ACCACAGTCCATGCCATCAC-3′, and *Gapdh* reverse 5′-CACCACCCTGTTGCTGTAGCC-3′.

### qPCR analysis

E17.5, and P0 heart tissues were isolated from *Snrk* WT (n = 3), KO (n = 3) and HET (n = 3) embryos. CM RNA was isolated from Control or *SNRK* knockdown Cardiomyocytes from three independent CM differentiations. Total RNAs were extracted from dissected tissues by TRIzol reagent followed by DNase I (Life Technologies) treatment for 2 h at 37°C. The DNA-free RNA was further purified using RNAeasy Mini kit (Qiagen). RNA concentrations were measured by Nanodrop (Thermo Scientific) and adjusted equally. SuperScript III (Life Sciences) was used for reverse transcription qPCR on 500 ng total RNA. qPCR analysis was used to assess *Snrk* knockdown in E17.5 hearts. Samples were assembled in DyNAmo™ HSSYBR® Green (Thermo Scientific) and run in a Bio-Rad real-time thermal cycler according to manufacturer's instructions. The expression levels were normalized to ribosomal protein large P0 (RPLP0). Primers: *Snrk* Exon 3 forward 5′-CGGGTCTCTTGCATACTCTG-3′, *Snrk* Exon 4 reverse 5′-ACACCAGCATGAAAAGGATC-3′, *Ribosomal Protein, Large P0 (Rplp0)* Exon 3 forward 5′-AGATTCGGGATATGCTGTTGGC-3′ and *Rplp0* Exon 4 reverse 5′-TCGGGTCCTAGACCAGTGTTC-3′. qPCR analysis of *SNRK* expression in hESC CM samples as well as all Microarray validations was assembled in TaqMan gene expression master mix (Life Technologies) and run in a Bio-Rad real-time thermal cycler according to manufacturer's instructions. Life Technologies Taqman Primers: *18S* Hs99999901_s1, *SNRK* Hs00299395_m1 and Mm00505252_m1; *peroxisomal trans-2-enoyl-CoA reductase (PECR)* Hs01032980_m1 *and* Mm01256685_m1; *ribose 5-phosphate isomerase A (RPIA)* Hs01107136_m1 and Mm00485790_m1; *acyl-CoA synthetase long-chain family member 4 (ACSL4)* Hs00244871_m1 and Mm00490331_m1; *fatty acid binding protein 3 (FABP3)* Hs00997360_m1 and Mm02342495_m1; *fatty acid binding protein 4 (FABP4)* Hs01086177_m1 and Mm00445878_m1; *glycerol kinase 2 (GYK)* Hs04235340_s1 and Mm00433896_m1; *acetyl-CoA carboxylase alpha (ACACA)* Hs01046047_m1 and Mm01304257_m1; *solute carrier family 22 member 5 (SLC22A5)* Hs00929869_m1 and Mm00441468_m1; *forkhead box O3 (FOXO3)* Hs04195365_s1 and Mm01185722_m1; *hypoxanthine guanine phosphoribosyl transferase (Hprt)* Mm00446968_m1. The gene expression levels in CM were normalized to *18S* and the gene expression levels in mouse hearts were normalized to *Hprt.*

### Microarray analysis

E17.5 heart tissues were isolated from WT (n = 3), and KO (n = 3) embryos and sent to Arraystar Inc, Rockville MD for RNA isolation and microarray analysis. RNA was isolated using TRIzol reagent (Life Technologies). Methodologies related to RNA labeling, array hybridization and data analysis are available upon request, and are also available directly from Arraystar Inc. GO analysis and Pathway analysis were performed using the standard enrichment computation method. Genes were selected using the following criteria: 1) upregulated or downregulated at least 1.5 fold and 2) *p-value*<0.5.

### Cryo-sectioning and immunofluorescence (IF) analysis

E17.5 embryos and P0 pups were fixed in 4% paraformaldehyde for 1 to 2 days at 4°C. After fixation, the samples were washed 3 times in PBS for 5 min each and then subjected to OCT embedding (Cossette and Misra, 2011). The samples were mounted for transverse sections for torsos or coronal sections for isolated hearts and subjected to 7–10 µm thick sectioning and placed on consecutive slides for multiple marker analysis. The sections were subjected to immunofluorescence analysis as previously described (Battle et al., 2006; Holtz and Misra, 2008). The following reagents were used: endothelial cell surface marker anti-platelet endothelial cell adhesion molecule-1 (PECAM-1; Life Technologies), smooth muscle marker anti-smooth muscle actin (SMA; Enzo Life Sciences), CM marker anti-homeobox protein NKX2.5 (Santa Cruz) and anti-cardiac troponin T (cTnT; Abcam), apoptosis maker anti-cleaved caspace 3 (CC3; Cell Signaling Technology), proliferation marker anti-phosphohistone H3 (PH3; Millipore), anti-SNRK antibody (ab) 1 (Abcam ab96762) anti-SNRK ab2 (Abcam ab94540) and anti-SNRK ab3 (Abgent AP7249c). Secondary antibodies used include: Alexa Fluor 488 (Life Technologies) and Alexa Fluor 568 (Life Technologies). The nucleus was stained with 4,6-Diamidino-2-Phenylindole (DAPI; Life Technologies). Images were captured using a Nikon Eclipse 90i microscope equipped with a Nikon C-1 Confocal and EZ C1 imaging software. Image processing was performed in Adobe Photoshop CS5.1.

### Whole mount heart measurements

Hearts were isolated from *Snrk* WT (n = 10) and *Snrk* KO (n = 10) E17.5 mouse embryos. Images were captured during diastole using a Zeiss microscope equipped with AxioCam MRc5 and AxioVision Rel. 4.8 imaging software. Image processing was performed in ImageJ. The area of the ventricle was measured by carefully drawing the circumference of the heart ventricles and using ImageJ software to calculate the total ventricle area. Measurement were normalized to the total weight of the embryo and graphed as the average of the percentage compared to wild type.

### Paraffin sectioning and staining

E17.5 and P0 neonate hearts and torsos were harvested and imaged followed by fixation with 10% zinc formalin for 1 to 2 days at 4°C. After fixation, tissues were washed 3 times in PBS for 5 min each and then subjected to Paraffin embedding. The tissues were cut into 4 to 7 µm thick sections and stained with Hematoxylin and Eosin (H&E) and Periodic Acid Schiff. Images were captured using a Zeiss microscope equipped with AxioCam MRc and AxioVision Rel. 4.8 imaging software. Image processing was performed in Adobe Photoshop CS5.1 and ImageJ.

### Oil Red O staining

E17.5, P0 neonate heart, torso, and isolated organs, and adult hearts were fixed in 4% Paraformaldehyde for 1 to 2 days at 4°C and then subjected to 7 to 10 µm cryosectioning followed by Oil Red O staining and hematoxylin staining. Images were captured using a Zeiss microscope equipped with AxioCam MRc and AxioVision Rel. 4.8 imaging software. Image processing was performed in Adobe Photoshop CS5.1 and ImageJ.

### Heart section quantification analysis

Periodic acid-Schiff (PAS), Oil Red O (ORO) and H&E stained sections of dissected or *in situ* hearts were scanned at 40× magnification using the Nanozoomer HT whole microscope slide scanning system (Hamamatsu, Japan) in the Pediatric BioBank and Analytical Tissue Core at the CRI and MCW. Three stained sections were analyzed per individual heart. The area data of the ventricle lumina were determined using the area tool in NDPView software (version 2.5), and transferred to an Excel worksheet for further analysis. Quantitative analysis for the P0 ventricle size was performed using the area tool in the NDPView software to determine the ventricle area. Quantitative analysis for PAS/glycogen and Oil Red O/lipid content in heart sections was performed using Visiomorph software (Visiopharm, Denmark). The software was trained to detect dark pink pixels for PAS, red pixels for ORO staining, blue pixels for unstained tissue, and white pixels for background. Linear Bayesian classification was used for segmentation into classes. The area values for the classes were transferred to an Excel worksheet, and the percentage of PAS or ORO content was calculated as [area pixels (PAS or ORO)/area pixels (PAS or ORO) + area tissue]*100.

### Blood plasma isolation

P0 neonates were anesthetized followed by decapitation. The blood was quickly collected and centrifuged at 14,000 rpm for 5 min. The supernatant containing the plasma was used for concentration analysis of triglyceride, total cholesterol, non-esterified free fatty acid and phospholipids. These test were conducted using colorimetric assays and analyzed on a plate reader at the University of Cincinnati Mouse Metabolic Phenotyping Center Lipid, Lipoprotein and Glucose Metabolism Core (OH, USA).

### Western blot (WB) analysis

E17.5 heart tissues were collected from *Snrk* wild-type (WT), KO and HET embryos, and the proteins were isolated using homogenization in RIPA buffer (Sigma) with complete mini ETDA-free protease inhibitor cocktail (Roche) and PhosStop phosphatase inhibitor (Roche) using a Qiagen TissueRuptor. Samples were incubated on ice for 20 min and cleared at 13,300 rpm at 4°C for 30 min. The protein concentration was determined using the BioRad DC protein assay followed by detection in a SpectraMax 340PC absorbance microplate reader. The Lowry quantification method was used to determine the protein concentration of the sample lysates. 30 µg of total protein were loaded to each well on a 12% Mini-PROTEAN TGX precast gel (BioRad) and subjected to SDS-PAGE. The proteins were transferred to polyvinylidene difluoride (PVDF) and immunoblotted. The following antibodies were used: anti-Tubulin (Sigma), anti-ACC (Cell Signaling Technology), anti-phospho ACC (pACC; Cell Signaling Technology), anti-AMPKα (Cell Signaling Technology), anti-phospho AMPKα (pAMPK; Cell Signaling Technology), anti-fatty acid synthase (FAS; Cell Signaling Technology), anti-phosphofructokinase 2 (PFK2; Santa Cruz), anti-phosphoPFK2 (pPFK2; Santa Cruz), anti-microtubule associated protein 1A/1B light chain 3 (LC3; Novus Biologicals); anti-phosphoenolpuruvate carboxykinase (PEPCK; Santa Cruz); anti-SNRK ab1 (Abcam ab96762); anti-Tubulin (Sigma), anti-rabbit horseradish peroxidase (HRP; Cell Signaling Technology) and anti-mouse HRP (Cell Signaling Technology).

### Co-immunoprecipitation

Neonatal and adult hearts were lysed with RIPA buffer (Sigma) containing complete mini EDTA-free protease inhibitor cocktail (Roche) and PhosStop phosphatase inhibitor (Roche) using a Qiagen TissueRuptor (Valencia, CA). Samples were incubated on ice for 20 min and cleared at 13,300 rpm at 4°C for 30 min. A portion of the resulting supernatants were reserved for total cell lysates, and equal amounts of the remaining total protein were immunoprecipitated using an anti-SNRK antibody (Abcam) with protein G agarose beads (Thermo Scientific). Immunoprecipitates were washed 3× with dilution buffer (10 mM Tris, 130 mM NaCl, 0.05% Triton X-100, 0.1% BSA, protease inhibitors). Additional washes with 50 mM Tris (pH 8.0) and Tris-Saline (10 mM Tris HCl, 140 mM NaCl) (pH 8.0) were also performed. Immunoprecipitates and total cell lysates were subjected to western blotting with the indicated antibodies: anti-SNRK antibody (Abcam), anti-Tubulin (Sigma) and anti-rabbit HRP (Cell Signaling Technology) and anti-mouse HRP (Cell Signaling Technology).

### Human cardiomyocyte system

Directed differentiation of CMs from human embryonic stem cells (hESCs) is described in previous studies (Lian et al., 2012; Lian et al., 2013). Briefly, hESCs maintained on matrigel in mTeSR1 were dissociated into single cells using Accutase (Life Technologies) at 37°C for 5 min and then were seeded onto a matrigel-coated 12-well plate at 1–2×10^5^ cell/cm^2^ in mTeSR1 supplemented with 5 µM Y-27632 (Tocris) for 24 h. After 24 h, fresh mTeSR1 medium was used every day to expand stem cells. When cells achieved a density of 5×10^5^ cell/cm^2^, cells were treated with 12 µM CHIR99021 (Selleckchem) in RPMI/B27 without insulin for 24 h (day 0 to day 1). The medium was changed to RPMI/B27 without insulin on day 1, followed by 5 µM IPW2 (Tocris) treatment on day 3. During the day 5 medium change IWP2 was removed. Cells were maintained in the RPMI/B27 starting from day 7, with the medium changed every 3 days.

### Lentiviral transduction of CMs and drug treatments

CMs from hESCs were dissociated into single cells with 0.25% Trypsin-EDTA (Life Technologies) at 37°C for 5 min, and then were spun down into aliquots of 1.5×10^6^ cells. Cells were resuspended in 30 µL concentrated lentivirus medium in the presence of 6 µg/mL polybrene (Sigma), and then incubated at room temperature for 10 min. At the end of incubation, 2 mL of DMEM/10% FBS + 5 µM Y-27632 (Tocris) was added and the suspension was transferred into a well of a 6-well plate pre-coated with Matrigel, and incubated at 37°C, 5% CO_2_ overnight. The medium was replaced with fresh DMEM/10% FBS on the second day of lentiviral infection. 2 days later, infected cells were selected and enriched for 3 days based on resistance to 1 µg/mL puromycin (Sigma). Two lentiviral constructs containing the empty vector shRNA Control (pGIPZ, Thermo Scientific) and the shRNA targeting human *SNRK* (Thermo Scientific) 5′-AGTGTGAAAGTCCAAGAGA-3′ were used to infect differentiated CMs. After selection, the CMs were treated with either DMSO (Sigma), 2 mM Metformin (Millipore) or 1 mM AICAR (Millipore) for 20–22 h. After treatment the cells were rinsed with PBS and the proteins were isolated as previously described.

### Cardiomyocyte autophagy study

After lentiviral infection and selection the culture medium was removed and replaced with serum free medium containing either water or 25 µM Choloroquine (CQ) for 4 hours. After treatment the cells were rinsed with PBS and the proteins were isolated as previously described.

### Metabolic flux study

The Seahorse XF-96 Flux Analyzer (Seahorse Bioscience Inc. Billerica, MA USA) was used to determine the metabolic profile of cardiomyocytes. 1.5×10^4^ cells per well were seeded in Roswell Park Memorial Institute (RPMI) assay medium without sodium bicarbonate in a V3-PET cell culture plate. The cells were then treated with 400 µM Palmitate or BSA (kit provided by Seahorse Bioscience) for 30 minutes prior to mitochondrial stress test analysis. Titrations were performed to determine the optimal concentrations of: oligomycin (1 µM) (Sigma–Aldrich), carbonyl cyanide 4-trifluoromethoxy-phenylhydrazone (FCCP) (1 µM) (Sigma–Aldrich), and Antimycin A (10 µM) (Sigma–Aldrich). After analysis, cardiomyocytes were lysed in 0.1% Triton X-100 and 10 µM Tris-HCl and quantified using the Bradford Protein Assay (Biorad). The metabolic flux data were normalized to total protein and analyzed for Basal Respiration, ATP Production and Maximal Respiration rate.

### Echocardiography and image analysis

Transthoracic echocardiography was performed in anesthetized (2% isoflurane) 6 month old litter matched males. An investigator (L.M.H.) who was blinded to the study groups performed the measurements and data analyses. Animals were studied with a commercially available echocardiographic system (Vivid 7, General Electric, Wauwatosa, WI), with an 11-MHz M12-L linear array transducer. Transthoracic echocardiography was performed from the cardiac short axis of the left ventricle at the papillary muscle level. Using the anatomical M-mode feature of the Vivid 7 echo, an M-mode display was generated from raw data 2D images with the line selected passing through the anterior and inferior segments. Ejection fraction % (EF) was measured using left ventricle end diastolic volume (LVEDV) and left ventricle end systolic volume (LVESV) using the formula EF = LVEDV−LVESV/LVEDV×100. Fractional shortening % (FS) was calculated with the formula: left ventricle end diastolic dimension (LVEDD)-left ventricle end systolic dimension (LVESD)/LVEDD×100. For every measurement, three consecutive heartbeats were measured, and the average used for analysis.

### Statistics

Student's unpaired t-test and one-way ANOVA was used for comparison analysis and the results are described as means (± standard error of the mean, SEM).

## RESULTS

### *Snrk* knockout (KO) mouse characterization

**Fig. 1.**
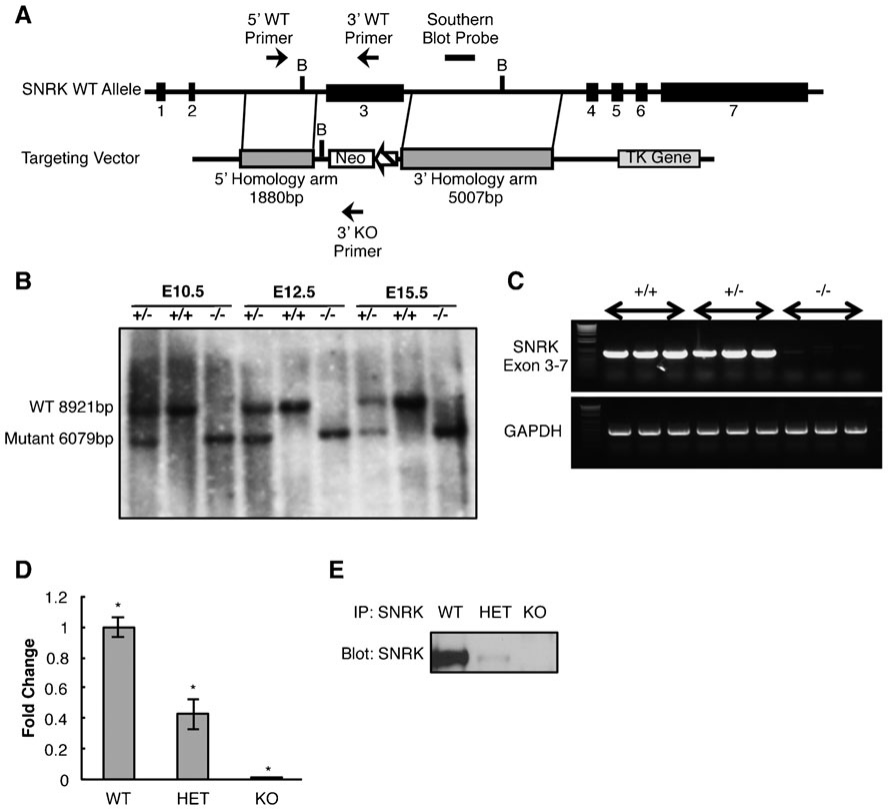
*Snrk* gene targeting and confirmation. (A) Schematic illustration of the strategy used to generate the global *Snrk* knockout (KO) mice. *Snrk* exon 3 was targeted and replaced with a neomycin cassette resulting in an *Snrk* KO allele. (B) Southern blot analysis of BamHI digested genomic DNA isolated from staged *Snrk* heterozygous (+/−) wild-type (+/+) and knockout (−/−) embryos at stage E10.5, E12.5 and E15.5 confirm successful targeting and generation of the *Snrk* KO allele. Wild-type allele will result in 8921 bp band, and the mutant targeted KO allele will results in a 6079 bp band. (C) *Snrk* KO embryos do not express *Snrk* by RT-PCR analysis of RNA isolated from E12.5 heterozygous (+/−) wild-type (+/+) and knockout (−/−) embryos using PCR primers that span *Snrk* Exon 3 to Exon 7 (719 bp) and control PCR primers for GAPDH (450 bp). (D) qPCR analysis confirms that *Snrk* RNA expression is decreased in *Snrk* HET and completely absent in *Snrk* KO E17.5 hearts. The results are the mean of the fold change of the Δ^2^CT ± SEM from three independent embryonic hearts for each genotype. The expression levels were normalized to RPLP0. * *p-value*<0.05. (E) SNRK protein expression is decreased in *Snrk* HET and completely absent in *Snrk* KO tissue. SNRK was immunoprecipitated using anti-SNRK antibody followed by western blot analysis with anti-SNRK. Results are representative of three independent experiments with equal protein loading.

To assess the role of *Snrk* in a mammalian model system, we have generated a global *Snrk* gene knockout in which exon 3 was replaced with a neomycin cassette using homologous recombination (Fig. 1A). *Snrk* exon 3 contains the ATG start site and the first 196 amino acids (aa) of the SNRK kinase domain. The kinase domain includes the predicted ATP binding site (aa15–45), the predicted kinase active site (aa135–147) (Kertesz et al., 2002), and the LKB1 phosphorylation site threonine 173 (Jaleel et al., 2005). Southern blot with a common genomic probe clearly showed one band (8921 bp) in E10.5, E12.5 and E15.5 *Snrk^+/+^* (WT) embryos, two bands (8921 bp and 6079 bp) in *Snrk^+/−^* (HET) and one band (6079 bp) in *Snrk^−/−^* (KO) embryos (Fig. 1B). The lower 6079 bp band indicates the altered genomic locus in *Snrk* HET and KO embryos. Loss of *Snrk* expression (exon 3) was confirmed using RT-PCR analysis with PCR primers spanning exons 3 to 7. Input RNA was isolated from E12.5 *Snrk* KO, *Snrk* HET and *Snrk* WT embryos (Fig. 1C). Additionally, qPCR validation was conducted on E17.5 mouse heart tissue from *Snrk* KO, HET and WT embryos (Fig. 1D). Primers specific to exons 3 to 4 showed an expected gradation in loss of *Snrk* mRNA with no detectable mRNA expression in *Snrk* KO embryos, and half in *Snrk* HET compared to the *Snrk* WT embryos. Loss of protein expression was confirmed using immunoprecipitation (IP) with SNRK-specific antibody1 (Ab1) followed by western blot (WB) analysis for SNRK in E17.5 mouse heart tissue from *Snrk* KO, HET and WT embryos (Fig. 1E). Similar to qPCR analysis there was no detectable expression in *Snrk* KO embryos and a substantial reduction of SNRK expression in *Snrk* HET embryos. Based on these results we conclude that we have generated an *Snrk* null mutant allele.

### Embryonic expression of SNRK

**Fig. 2.**
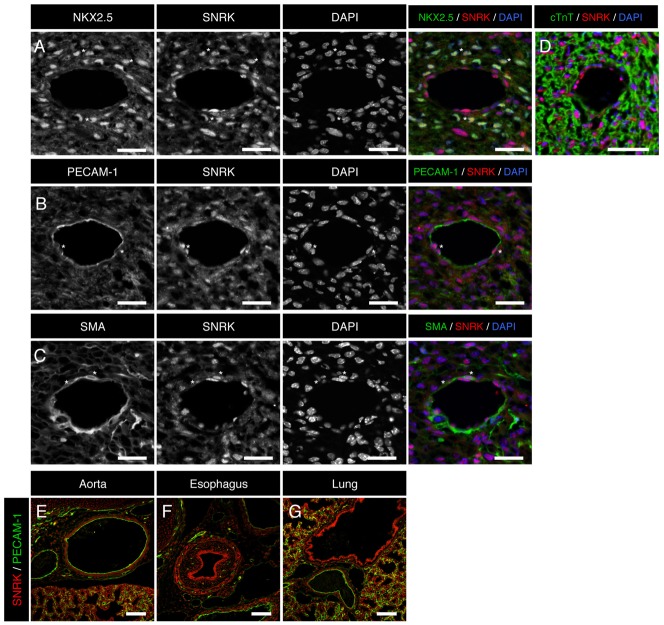
SNRK is expressed in cardiac and vascular cell types as well as endoderm derived tissues. Immunofluorescence analysis of transverse-sectioned E17.5 WT embryos is depicted. (A–D) Cardiac, (E) aorta, (F) esophagus and (G) lung. Sections were stained with SNRK (red), nuclear DNA marker DAPI (blue), cardiac markers NKX2.5 or cTnT (green), endothelial cell marker PECAM-1 (green) and smooth muscle cell marker SMA (green). Results are representative of three independent experiments. *Demarks examples of co-expressing cells. Scale bars: 25 µm (A–C), 50 µm (D), 100 µm (E–G).

As a prelude to the phenotypic characterization, we investigated SNRK protein expression using immunofluorescence (IF) microscopy technique on mouse embryo sections. *Snrk* has been previously shown by in situ hybridization to be expressed in the neural epithelial, the gut endoderm, aortic endothelial cells as well as CMs and endocardium (Kertesz et al., 2002), and WB analysis has confirmed SNRK expression in a wide range of adult tissues such as heart, brain, intestine and adipocytes (Li et al., 2013). At E17.5 SNRK protein expression was detected by IF using three different antibodies (supplementary material Fig. S1). SNRK ab1 was used for subsequent analysis, and protein expression is observed in a wide range of tissues including; aortic endothelial cells, aortic smooth muscle cells, (Fig. 2E) the esophagus epithelium (Fig. 2F), lung epithelium (Fig. 2G), as well as in several heart cell types such as, the coronary endothelial, and smooth muscle cells, the endocardium and the CMs (Fig. 2A–D). The localization of SNRK is observed in both nuclear and cytoplasmic compartments of smooth muscle cells, endothelial cells and CMs (Fig. 2A–D). The expression analysis suggests that SNRK is widely expressed in all three tissues that comprise of the germ layers ectoderm, endoderm and mesoderm.

### Loss of *Snrk* results in neonatal lethality with enlarged hearts

To determine whether the loss of *Snrk* resulted in lethality, embryos and neonates at various stages were collected and genotyped (supplementary material Table S1). *Snrk* HET breeding mating pair results in pups with the expected Mendelian ratio 1:2:1 (25%:50%:25%) throughout embryonic development. At birth all neonates, WT, HET and KO pups appear phenotypically normal (supplementary material Fig. S2), however within 12–24 h, we observed a loss of the expected Mendelian ratio (P1 38.89%:55.56%:5.56%, P2 35.00%:65.55%:0%), and observed neonatal death occurring in all of the *Snrk* KO pups within 24 h with no pups surviving beyond 24 h. These results along with the expression analysis data suggest that *Snrk* is important for neonatal survival.

**Fig. 3.**
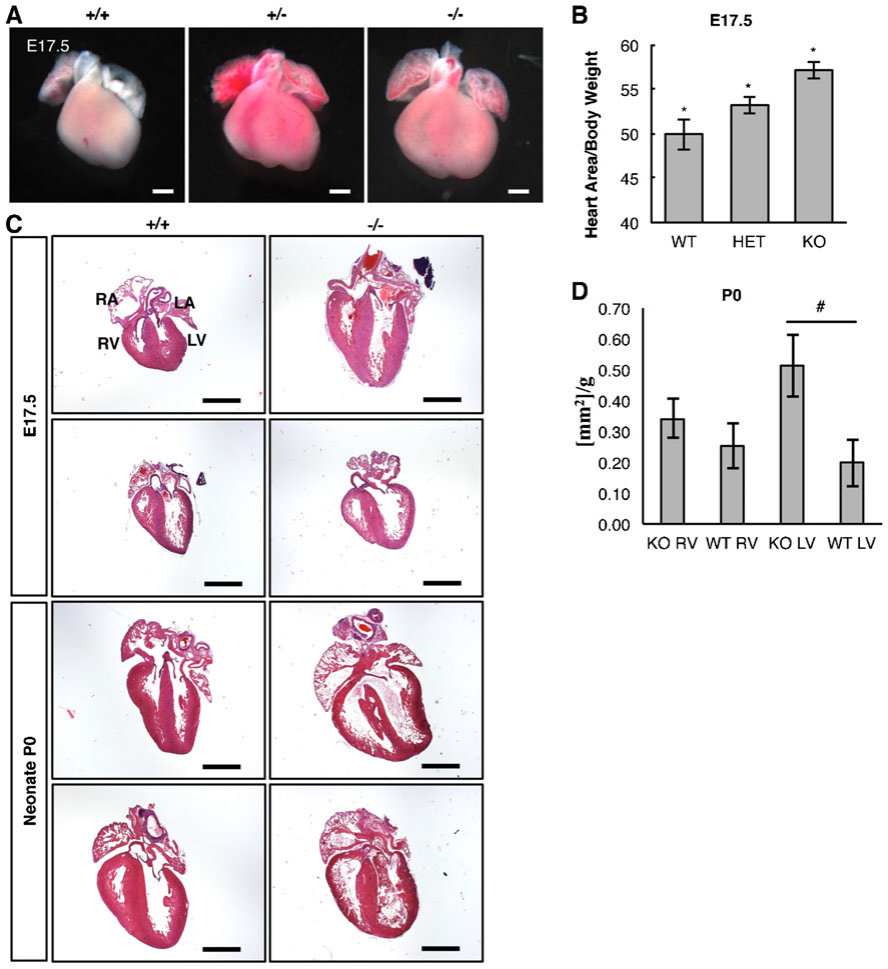
Loss of *Snrk* results in enlarged E17.5 and Neonate (P0) hearts. (A) Photomicrographs of litter matched E17.5 hearts isolated from *Snrk* WT (+/+), HET (+/−), and KO (−/−) embryos. (B) E17.5 quantification of the heart size was conducted by calculating the surface area of the ventricles shown in panel A divided by the total body weight of each embryo. The results are the mean ± SEM from ten independent E17.5 embryonic hearts for each genotype. KO 57.11, WT 49.91, Het 53.11, *p* = 0.00122 (KO vs. WT), *p* = 0.020 (KO vs. HET). (C) Photomicrograph of E17.5 and neonate coronal-sectioned hearts. Sectioned were subjected to Hematoxylin and Eosin (H&E) staining. (D) Neonate P0 quantification of the individual ventricle areas of H&E stained hearts normalized to body weight. The results are the mean ± SEM from 3 WT and 4 KO P0 hearts. KO RV 0.34, WT RV 25, KO LV 0.51, WT LV 0.20, *p* = 0.10. RA, right atria; LA, left atria; RV, right ventricle; LV, left ventricle. Scale bars: 500 µm (A), 1 mm (C). * *p-value*<0.05, # 0.05<*p-value*<0.10.

To study the phenotypes resulting from the loss of *Snrk* we isolated tissues at P0 and E17.5. During necropsy and tissue sectioning of neonatal hearts, we consistently observed larger hearts for *Snrk* KO mice. Quantitation of E17.5 *Snrk* KO mouse heart showed significant increase in size than *Snrk* WT or HET embryos based on surface area normalized to body weight (Fig. 3A,B). Quantification of P0 *Snrk* KO mouse heart showed significant increase in the size of the left ventricle (LV) based on ventricle area analysis normalized to body weight of Hematoxylin and Eosin (H&E) stained coronal heart sections (Fig. 3C,D). The area of the right ventricle (RV) did not show a significant increase in size (Fig. 3C–D). To determine if increased proliferation or decreased apoptosis accounts for the increased size of the embryonic hearts, we conducted IF analysis for phosphohistone H3 (PH3) or cleaved caspase 3 (CC3) respectively on E17.5 heart sections, and observed no differences in either markers between *Snrk* KO or WT hearts (data not shown). These results suggest that the increased heart size in *Snrk* KO is not the result of increased proliferation or decreased apoptosis of cells contributing to the cardiac tissue.

### Microarray analysis of *Snrk* knockout hearts reveals metabolic dysregulation

**Fig. 4.**
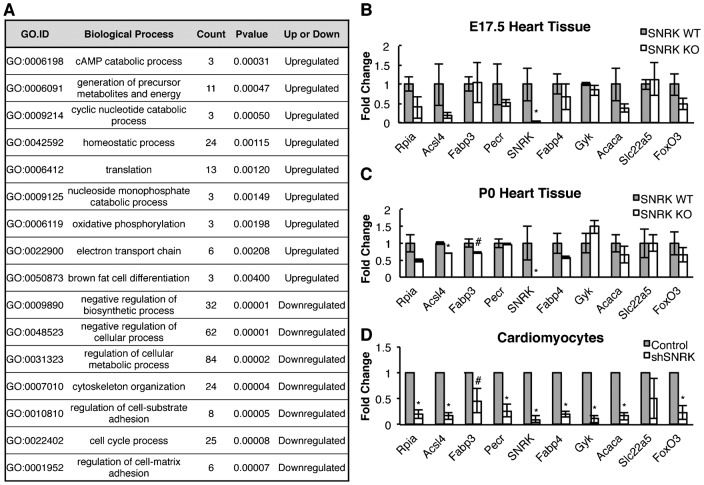
Gene ontology data and microarray validation of metabolism genes. (A) Gene ontology (GO) terms for biological processes that over represented in SNRK WT and SNRK KO microarray data sets. Fold change analysis obtained by TaqMan qPCR analysis from mRNA isolated from E17.5 (B) and Neonate (C) SNRK WT and KO hearts (n = 3 for each genotype) and (D) *SNRK* knockdown in hESC-derived CMs infected with empty vector shRNA control (Control) lentivirus and *SNRK* shRNA lentivirus (shSNRK) (n = 3 from three independent cardiomyocyte infections). The results are the mean of the fold change ± SEM. * *p-value*<0.05, # 0.05<*p-value*<0.10.

To gain insight into the molecular mechanism of the increased heart size, we performed microarray expression analysis on E17.5 *Snrk* WT, HET and KO mouse embryonic hearts, a developmental stage that was close enough to birth to avoid implication associated with neonatal lethality. Total RNA was isolated from three independent embryonic hearts per genotype, and RNA hybridized using Nimbelgene/Roche microarrays (ArrayStar Inc.). A total of 839 genes with a *p-value*<0.05 and a fold change cut off of ±1.5 showed differential expression in *Snrk* KO compared to *Snrk* WT; a total of 541 genes with a *p-value*<0.05 and a fold change cut off of ±1.5 showed differential expression in *Snrk* HET compared to *Snrk* WT; and 152 of the same genes with a *p-value*<0.05 and a fold change cut off of ±1.5 showed differential expression in both *Snrk* KO and HET compared to *Snrk* WT hearts (supplementary material Table S2). Gene ontology (GO) analysis was performed on the array results to determine the enrichment score of differentially expressed genes. The biological processes that displayed high enrichment which were *upregulated* in the *Snrk* KO include: cyclic AMP catabolic processes, generation of precursor metabolites and energy, cyclic nucleotide catabolic process, homeostatic process, translation, nucleoside monophosphate catabolic process, oxidative phosphorylation, electron transport chain, and brown fat cell differentiation (Fig. 4A). The biological processes that displayed high enrichment scores which were *downregulated* in the *Snrk* KO include: negative regulation of biosynthetic and cellular processes, regulation of cellular metabolic process, cytoskeleton organization, regulation of cell-substrate adhesion and cell-matrix adhesion, and cell cycle process (Fig. 4A). To validate the expression changes identified in the microarray, TaqMan qPCR analysis was conducted on select groups of genes involved in metabolism using cDNA isolated from WT and KO E17.5, neonates and *Snrk* knockdown CM (Fig. 4B–D). Taken together, these results suggest that *Snrk* KO hearts show dysregulated metabolic regulation.

### Loss of *Snrk* results in metabolic abnormalities in cardiac tissue

**Fig. 5.**
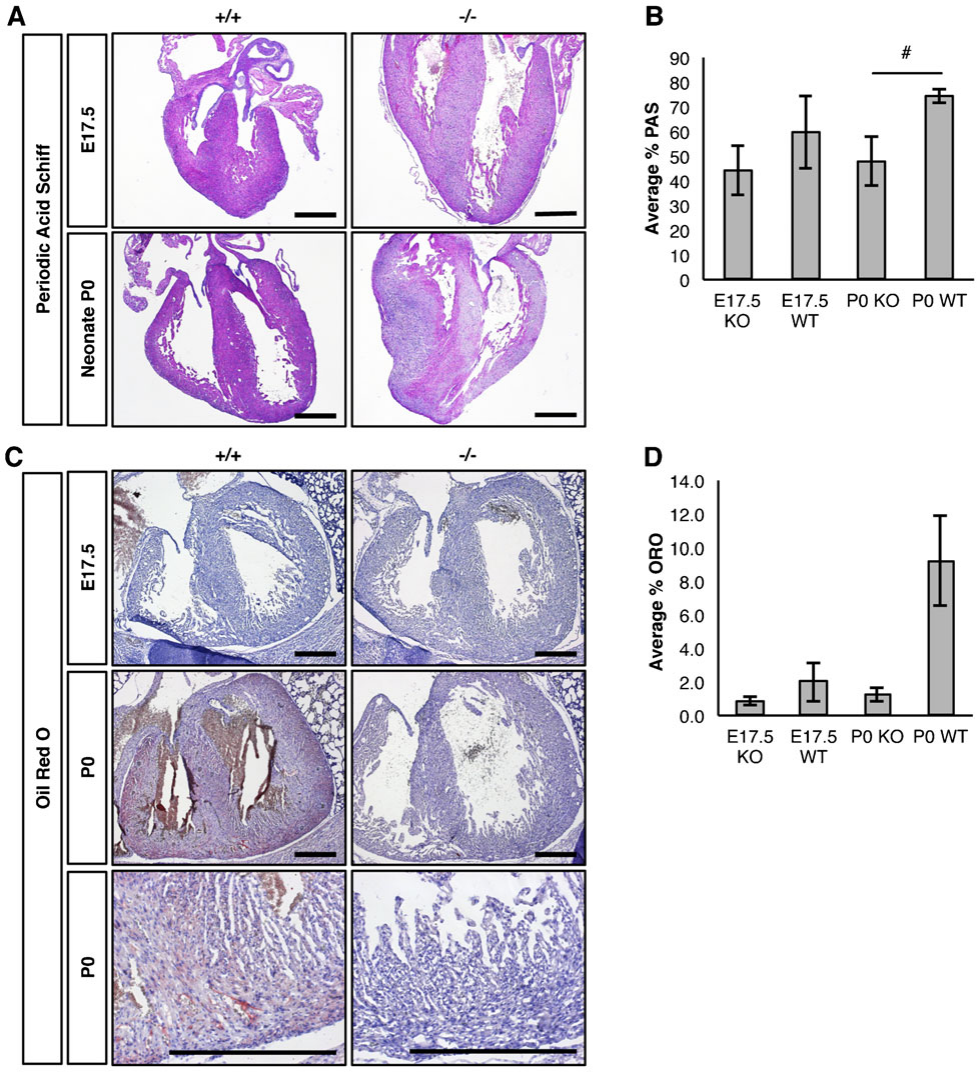
Loss of *Snrk* results in glycogen and lipid defects in mouse hearts. (A) Photomicrograph of E17.5 and P0 coronal-sectioned hearts. Sectioned were subjected to PAS staining. Pink color demarks the presence of stored glycogen and purple/blue color demarks the absence of stored glycogen. Results are representative of three independent experiments. (B) Quantification of the percentage of PAS staining was conducted by calculating the total area of purple/blue color divided by the total area of the heart. The results are the mean ± SEM from 3 WT and 5 KO independent E17.5 embryonic hearts and 3 WT and 4 KO P0 hearts. E17.5 KO 44.15%, WT 59.73%, *p* = 0.39; P0 KO 48.08%, WT 74.46, *p* = 0.07. (C) Photomicrograph of *Snrk* E17.5 and neonate transverse sectioned mice. Sections containing the heart were subjected to ORO staining followed by hematoxylin staining. Pink/red blush demarks the presence of lipids and blue demarks the hematoxylin stained nuclei. (D) Quantification of the percentage of ORO staining was conducted by calculating the amount of red ORO stain divided by the total area of the heart. The results are the mean ± SEM from 3 WT and 3 KO independent E17.5 embryonic hearts and 6 WT and 6 KO P0 hearts. E17.5 KO 0.987%, WT 2.01% *p* = 0.4; P0 KO 1.20%, WT 9.20 *p* = 0.24. Scale bars: 500 µm. # 0.05<*p-value*<0.10.

We focused next on the glucose and lipid utilization by the heart at E17.5 and P0 stages. We examined for the presence of stored glycogen and lipids in E17.5 and P0 sectioned hearts and embryos. Neonatal hearts were isolated from pups that contained milk spots, phenotypically looked normal and did not show any signs of distress such as changes in skin color. Histological analysis of *Snrk* KO embryonic neonate hearts (Fig. 5A,B) showed a large decrease in Periodic Acid Schiff (PAS) staining, a marker for tissue glycogen levels. Analysis of neutral lipids using Oil Red O (ORO) staining in neonatal hearts identified a complete absence of ORO staining in *Snrk* KO neonates compared to *Snrk* WT neonates (Fig. 5C,D). However, we did not observe changes in ORO staining in E17.5 *Snrk* WT or KO heart sections. ORO staining was observed in other tissues such as brown adipose tissue in *Snrk* WT and *Snrk* KO embryos and neonates (data not shown). We also examined the circulating lipids in blood plasma of litter-matched neonate *Snrk* WT and KO pups to determine whether the loss of ORO staining in the heart is a result of changes in circulating lipids (supplementary material Fig. S3). Indeed, analysis of non-esterified free fatty acid (NEFA) and esterified fatty acids or triglycerides (TGs) showed a significant decrease in *Snrk* KO pups (supplementary material Fig. S3). A significant increase in plasma cholesterol was also noted in *Snrk* KO pups, and no significant difference was observed in plasma phospholipids in *Snrk* KO or WT pups (supplementary material Fig. S3B).

**Fig. 6.**
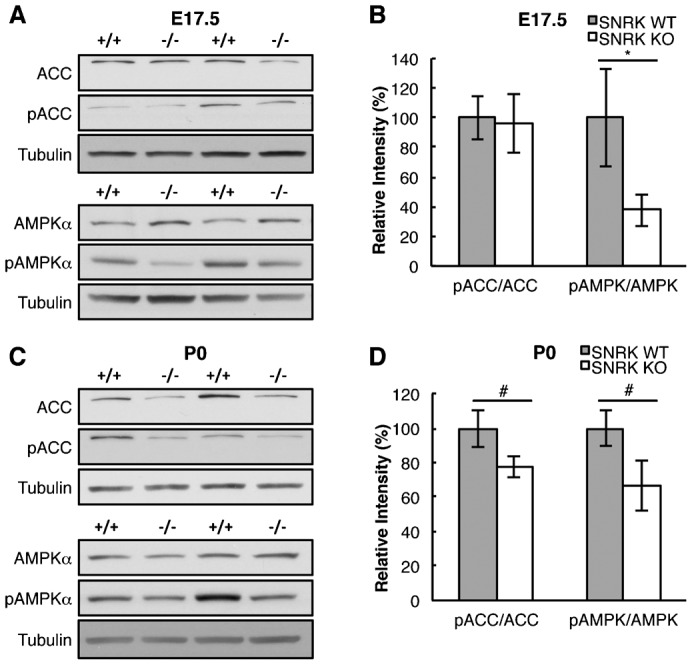
Loss of *Snrk* results in cellular metabolic defects. (A,B) The expression of ACC, pACC, AMPKα and pAMPKα in E17.5 heart lysates from *Snrk* WT (+/+) and KO (−/−) hearts was determined by western blotting followed by densitometry quantitation. The results are the mean ± SEM of the ratio of the phosphorylated protein normalized to the total protein. (C,D) The expression of ACC, pACC, AMPKα and pAMPKα in neonate heart lysates from *Snrk* WT and KO hearts was determined by western blotting followed by densitometry quantitation using tubulin as the loading control. The results are the mean ± SEM of the ratio of the phosphorylated protein normalized to the total protein. E17.5 pACC/ACC ratio: WT 100%, KO 95.61%, *p* = 0.978, n = 8WT, 7KO and E17.5 pAMPK/AMPK ratio: WT 100%, KO 37.55%, *p* = 0.038, n = 9. P0 pACC/ACC ratio: WT 100%, KO 77.24%, *p* = 0.102 n = 10 and P0 pAMPK/AMPK ratio: WT 100%, KO 66.44%, *p* = 0.074, n = 10. * *p-value*<0.05, # 0.05<*p-value*<0.10.

Western blot analysis of E17.5 and P0 neonatal heart tissue identified a decrease in the ratio of pACC, a common marker of cellular FAO in neonates (Fig. 6C,D). There was no significant difference in the ratio of pACC in E17.5 hearts (Fig. 6A,B). We also investigated for changes in the activation of AMPK via phosphorylation of AMPKα subunit. In E17.5 there was a significant decrease in pAMPKα levels (Fig. 6B), and a significant change in pAMPKα levels in P0 neonates (Fig. 6D).

To determine if other metabolic pathways are affected by the loss of *Snrk*, we examined the expression levels of the fatty acid synthesis marker fatty acid synthase (FAS), the gluconeogenesis marker phosphoenolpyruvate carboxykinase (PEPCK), the glycolysis marker and direct AMPK target phosphorylated phosphofructokinase 2 (pPFK2) (supplementary material Fig. S4), and did not observe a statistical difference between *Snrk* KO and WT hearts. Circulating lipid defects may also result from defects in intestinal lipid absorption or liver secretion. We performed ORO staining on liver and intestine sections of neonatal *Snrk* WT and KO pups, which showed reduced staining patterns in *Snrk* KO compared to *Snrk* WT littermates (supplementary material Fig. S5) thus suggesting defects in intestinal and liver lipid absorption as well. Collectively, these results suggest that the global loss of *Snrk* results in defects associated with cardiac tissue energy sources, and other organs as well, which could result in cardiac failure and cause neonatal lethality.

### *SNRK* knockdown in human CMs alters FAO pathway

**Fig. 7.**
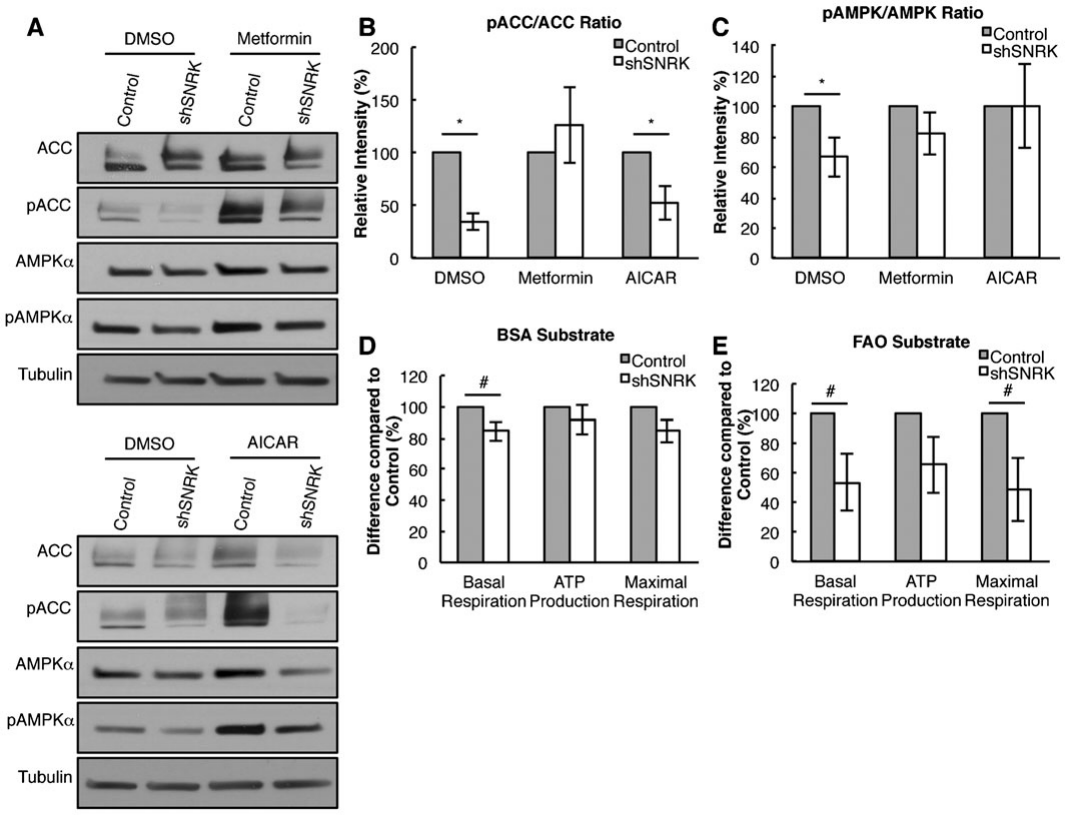
Cardiomyocyte-specific loss of *SNRK* results in cellular metabolic defects in vitro. The expression of ACC, pACC, AMPKα and pAMPKα in hESC-derived CMs infected with empty vector shRNA control (Control) lentivirus and *SNRK* shRNA lentivirus (shSNRK) was determined by western blotting (A) followed by densitometry quantitation (B,C). The CMs were treated with either DMSO, 2 mM Metformin or 1 mM AICAR. The results are the mean ± SEM of the ratio of the phosphorylated protein normalized to the total protein. pACC/ACC ratio: DMSO Control 100%, shSNRK 35.03%, *p* = 0.0002, n = 4; Metformin Control 100%, shSNRK 126.27%, *p* = 0.5, n = 4; AICAR Control 100%, shSNRK 52.41%, *p* = 0.017, n = 5. pAMPK/AMPK ratio: DMSO Control 100%, shSNRK 66.40%, *p* = 0.036, n = 4; Metformin Control 100%, shSNRK 82.29%, *p* = 0.261, n = 4; AICAR Control 100%, shSNRK 100.63%, *p* = 0.983, n = 4. (D,E) *SNRK* knockdown in CM results in a decrease in the oxygen consumption rate (OCR) displayed as Basal respiration, ATP production and Maximal Respiration. The CM OCR rate was assessed in basal medium containing bovine serum albumin (BSA) (Basal Respiration Control 100%, shSNRK 84.71%, *p* = 0.074, n = 3; ATP Production Control 100%, shSNRK 91.57%, *p* = 0.402, n = 3; Maximal Control 100%, shSNRK 84.69%, *p* = 0.103, n = 3) (D) or basal medium containing the FAO substrate (BSA-Palmitate) (Basal Control 100%, shSNRK 53.60%, *p* = 0.077, n = 3; ATP Production Control 100%, shSNRK 65.64%, *p* = 0.146, n = 3; Maximal Control 100%, shSNRK 48.30%, *p* = 0.073, n = 3) (E). The results are the mean ± SEM of the percentage of the OCR rate normalized to total protein. *p-value<0.05, # 0.05<*p-value*<0.10.

To confirm the in vivo findings in cardiac tissue, we used lentiviral shRNA for *SNRK* (shSNRK) to knock down *SNRK* expression in CMs differentiated from hESCs. Lentivirus expressing empty vector shRNA control (Control) and shSNRK were able to infect cultured CMs and confirmation of *SNRK* knockdown was assessed using qPCR analysis (Fig. 4D; supplementary material Fig. S6). We next investigated pACC and pAMPKα proteins in the *SNRK* knockdown CMs. Similar to the *Snrk* KO hearts (Fig. 6), we observed that ratios for both pACC and pAMPKα was significantly reduced in *SNRK* knockdown CMs (Fig. 7). To elucidate the mechanism of SNRK function in cardiac metabolism, we next investigated whether the indirect AMPK activator Metformin and the direct AMPK activator AICAR are able to rescue the decreased pACC and pAMPKα phenotype observed in shSNRK knockdown CMs (Fig. 7). Both Metformin and AICAR were able to rescue the pAMPKα decrease (Fig. 7C) observed in shSNRK CMs, but only Metformin was able to rescue the pACC phenotype (Fig. 7B) suggesting that SNRK independent of AMPK is required for normal ACC phosphorylation. We also examined the autophagy marker microtubule-associated protein 1A/1B-light chain 3 (LC3) in neonate hearts as well as in *SNRK* knockdown in hESC differentiated CM (supplementary material Fig. S4). We did not observe significant change in the LC3-II/LC3-I ratio in the neonate heart lysates (supplementary material Fig. S4C,D). Further, when autophagy was induced in cultured CM with Choloroquine (CQ) (supplementary material Fig. S4E,F) we did not observe any change in the control or *SNRK* shRNA knocked down CMs.

We next compared the metabolic flux between control shRNA and *SNRK* shRNA knockdown CMs using the Seahorse Bioscience Metabolic Flux Analyzer (Fig. 7D). Knockdown of *SNRK* resulted in a significant decrease in the basal respiration (Fig. 7D). When CMs were provided the FAO substrate BSA-Palmitate there was a significant decrease in the basal respiration rate and the maximal respiration compared to the control CMs (Fig. 7E), which indicates that SNRK is required for normal BSA-Palmitate substrate availability. We did observe a trend towards decreased FAO-ATP production (*p* = 0.145), which suggests that SNRK may also play a role in mitochondrial function (ATP production). These data together with the *Snrk* KO heart data suggest that loss of *SNRK* in CMs contributes intrinsically to the pACC-pAMPK signaling pathway responsible for FAO.

### Conditional loss of *Snrk* in endothelial cells alters cardiac FAO pathway

**Fig. 8.**
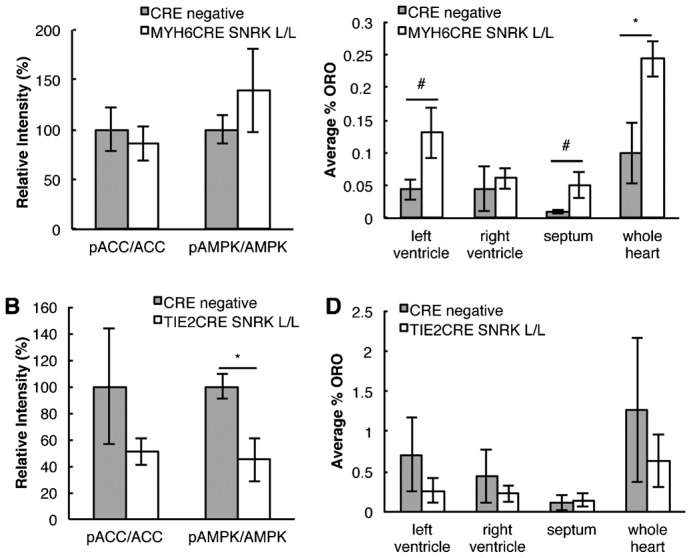
Conditional loss of *Snrk* in endothelial cells but not CMs results in metabolic abnormalities in vivo. (A) The expression of ACC, pACC, AMPKα and pAMPKα in neonate heart lysates from CRE negative and MYH6CRE-SNRK L/L was determined by western blotting followed by densitometry quantitation. The results are the mean ± SEM of the ratio of the phosphorylated protein normalized to the total protein (pACC/ACC ratio: WT 100%, cKO 85.72%, *p* = 0.62, n = 3 and pAMPK/AMPK ratio: WT 100%, cKO 139.36%, *p* = 0.394, n = 3). (B) The expression of ACC, pACC, AMPKα and pAMPKα in neonate heart lysates from CRE negative and TIE2CRE-SNRK L/L was determined by western blotting followed by densitometry quantitation. The results are the mean ± SEM of the ratio of the phosphorylated protein normalized to the total protein (pACC/ACC ratio: WT 100%, cKO 42.7%, *p* = 0.31, n = 6 and pAMPK/AMPK ratio: WT 100%, cKO 44.8%, *p* = 0.04, n = 3). (C,D) Quantification of the percentage of ORO staining was conducted by calculating the amount of red ORO stain divided by the total area of the heart. The results are the mean ± SEM from three neonate hearts for each genotype. Cardiac specific: WT LV 0.04%, RV 0.05%, Septum 0.01%, whole heart 0.10%; cKO LV 0.13% *p* = 0.10, RV 0.06%, Septum 0.05% *p* = 0.10, whole heart 0.24% *p* = 0.05. Endothelial specific: WT LV 0.71%, RV 0.45%, Septum 0.12%, whole heart 1.27%; cKO LV 0.26%, RV 0.23%, Septum 0.14%, whole heart 0.64%. * *p-value*<0.05, # 0.05<*p-value*<0.10.

To assess the cells in the cardiac tissue that are responsible for the observed cardiac metabolic defects in *Snrk* global KO mice, we generated an *Snrk* conditional deletion mouse line (*Snrk* LoxP) (supplementary material Fig. S7). The endothelial specific cre recombinase mouse line TIE2CRE (Kisanuki et al., 2001) and the CM-specific cre recombinase mouse line MYH6CRE (Agah et al., 1997) were introduced into the *Snrk* LoxP mouse line to generate an endothelial-specific *Snrk* KO or a CM-specific *Snrk* KO. The conditional knockout (cKO) lines were validated for *Snrk* genomic locus modification (supplementary material Fig. S7B), and for loss of SNRK protein expression (supplementary material Fig. S7C,D). The CM-specific loss of *Snrk* did not result in a net change in pAMPKα and pACC levels in total heart lysates. This result indicates that CM expression of *Snrk* is not required for normal pAMPKα and pACC levels in vivo or that it contributes to a small fraction of pAMPKα and pACC in the heart, and that any change in CM protein phosphorylation is masked by SNRK expression in other cell types, such as endothelial cells (Fig. 8A). In support, the endothelial-specific loss of *Snrk* resulted in significant decrease in pAMPKα and pACC levels in heart lysates. These results indicate that endothelial *Snrk* contributes to the majority of cardiac FAO (Fig. 8B). To assess whether there is a lipid absorption defect in neonatal hearts, we examined the ORO lipid staining in cKO hearts (Fig. 8C,D). CM-specific loss of *Snrk* resulted in a significant increase in ORO accumulation in the neonate hearts and the endothelial-specific loss of *Snrk* resulted in a decrease in ORO absorption that was not significant. We did not observe any neonatal lethality in CM-specific or endothelial specific cKO mice (supplementary material Table S3). Conditional KO studies in mice, and in vitro CMs data collectively suggest that *Snrk* participates in the regulation of cardiac FAO signaling, through a cross-talk mechanism between CM and endothelial cells.

### CMs-specific loss of *Snrk* in vivo results in cardiac functional defects and adult lethality

**Fig. 9.**
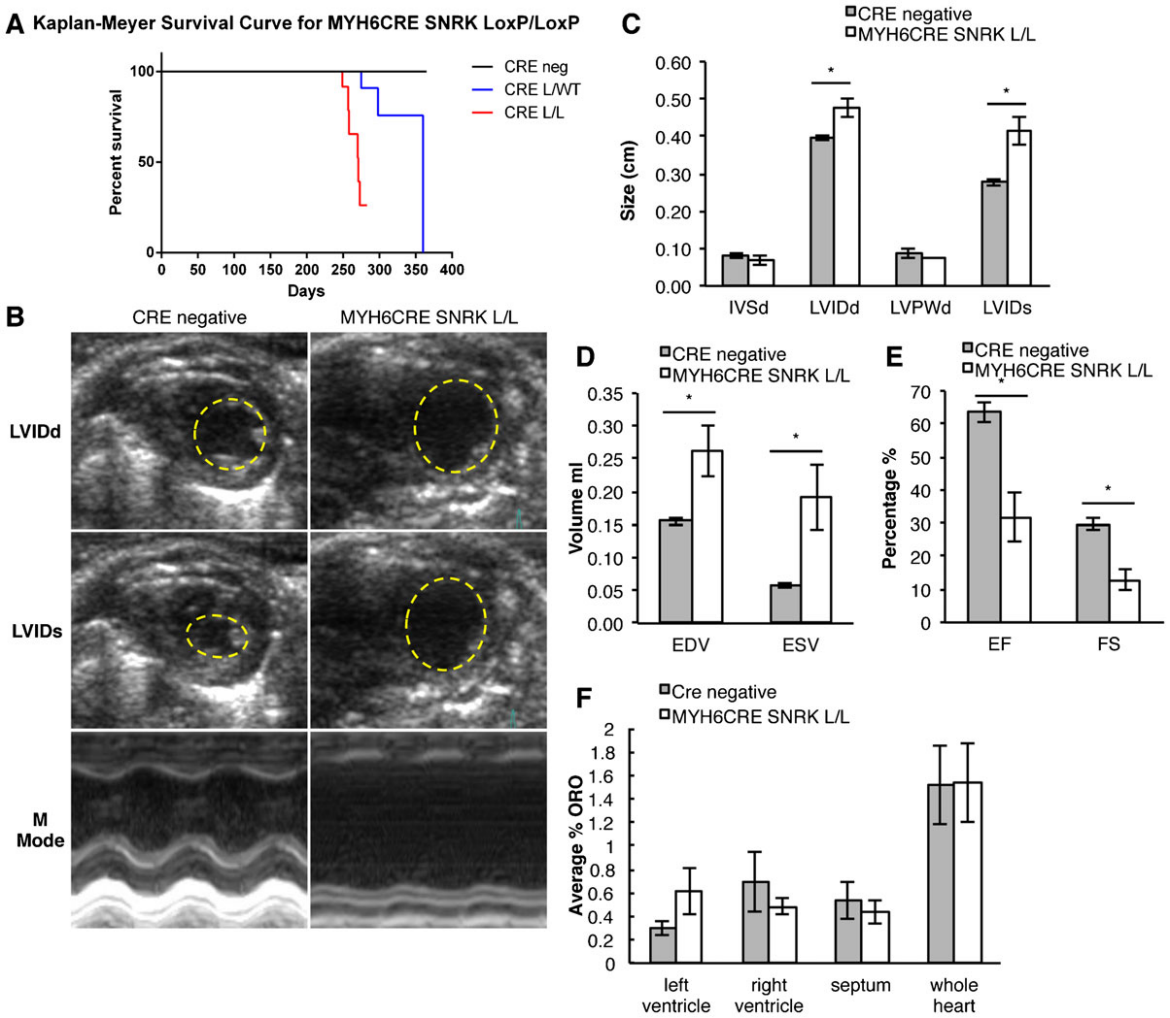
Conditional loss of *Snrk* in cardiomyocytes results in cardiac functional defects and adult lethality. (A) Kaplan–Meyer survival curve for CRE negative (n = 24), MYH6CRE SNRK LoxP/LoxP (CRE L/L, n = 24) and MYH6CRE SNRK LoxP/WT (CRE L/WT, n = 18) mice. (B) Representative ultrasound images from 6 month old adult male mice of the left ventricular internal diameter in end diastole (LVIDd), left ventricular inner diameter in end systole (LVIDs) and M Mode images. (C) The LV internal dimensions are increased at end-diastolic (LVIDd) and end-systolic (LVIDs) in the MYH6 cKO mice compared to the control (Cre negative: LVIDd 0.40 cm, LVIDs 0.28 cm; LoxP/LoxP: LVIDd 0.48 cm *p* = 0.018, LVIDs 0.42 cm *p* = 0.012, n = 4). Diastolic wall thickness of the intraventucular septum thickness in diastole (IVSd) and left ventricular posterior wall thickness in diastole (LVPWd) is unchanged between mice (n = 4). (D) End-diastolic volume (EDV) and end-systolic volume (ESV) are increased in MYH6 (n = 4) vs control hearts (Cre negative: EDV 0.16 ml, ESV 0.06 ml; LoxP/LoxP: EDV 0.26 ml *p* = 0.036, ESV 0.19 *p* = 0.037 n = 4). (E) MYH6 cKO hearts have decreased left ventricular systolic function shown by a decrease in the percent ejection fraction (EF) and fractional shortening (FS) (Cre negative: EF 63.48%, FS 29.67%; LoxP/LoxP: EF 31.63% *p* = 0.007, FS 12.83%, *p* = 0.004, n = 4). (F) Quantification of the percentage of ORO staining was conducted by calculating the amount of red ORO stain divided by the total area of the heart. The results are the mean ± SEM from four WT and three MYH6 cKO adult hearts (WT LV 0.29%, RV 0.69%, Septum 0.53%, whole heart 1.52%, cKO LV 0.61%, RV 0.48%, Septum 0.43%, whole heart 1.53%). Yellow dashed circle demarks the endocardial border of the left ventricle. * *p-value*<0.05, # 0.05<*p-value*<0.10.

To determine whether the loss of *Snrk* in CMs results in lethality in adult, we analyzed the survival of MYH6CRE *Snrk* LoxP/LoxP, MYH6CRE *Snrk* LoxP/WT and cre negative mice (Fig. 9A). Homozygous loss of *Snrk* (LoxP/LoxP) results in lethality between 8–10 months of age and heterozygous loss of *Snrk* (LoxP/WT) showed lethality shortly after one year of age. To assess the cause of adult lethality we examine whether the LoxP/LoxP mice have cardiac functional abnormalities. Adult (6 month old) MYH6CRE *Snrk* LoxP/LoxP and cre negative litter matched males were subjected to echocardiographic analysis. The left ventricle inner diameter (LVID) was significantly enlarged in systole (s) and diastole (d) in the MYH6CRE *Snrk* LoxP/LoxP mice compared to the cre negative littermates (Fig. 9B,C). Furthermore, in the MYH6CRE *Snrk* LoxP/LoxP mice there was a significant increase in end diastolic (EDV) and end systolic volume (ESV). The increase in left ventricular dimensions and volumes are indicators of adverse cardiac remodeling. In support, the mice also had a significant decrease in the ejection fraction (EF) and the fractional shortening (FS), measurements of left ventricular systolic function (Fig. 9D,E). We did not observe any changes in stroke volume, left ventricle mass or heart rate (data not shown). These observations suggest that *Snrk* is critically important for normal adult cardiac function.

## DISCUSSION

The ATP/AMP ratio is critical for cellular metabolic homeostasis, and alteration in this ratio affects cellular metabolic sensors such as AMPK. AMPK is a multi-subunit enzyme that participates in various signaling cascades related to metabolism, and the regulation of this process is complex and highly regulated (Nagendran et al., 2013). In this study, we have identified one member of the AMPK family namely SNRK, which is a critical regulator of cardiac energy homeostasis during cardiovascular development.

*Snrk* was first identified in 3T3-L1 differentiated adipocyte cells (Becker et al., 1996), and subsequently was shown to be expressed in a host of other tissues in mice and humans including white and brown adipose tissue (WAT and BAT), small intestine, heart, brain and liver (Li et al., 2013), tissues with high metabolic needs. Compared to *Ampkα1* and *Ampkα2*, *Snrk* expression in WAT, BAT, heart and brain is significantly higher (Li et al., 2013) implying that in these tissues, SNRK may provide the major metabolic regulatory function. Supporting this argument is the recent report that demonstrates *Snrk*'s involvement in adipocyte function and inflammation (Li et al., 2013). To date, *Snrk*'s function during embryonic mammalian development has not been investigated. In mice, *Snrk* mRNA is expressed at E8.0 in the hindbrain and primitive gut, and at E10.5 in the neural tube, aorta, and heart (endocardium and myocardium) (Kertesz et al., 2002). Our data here show that the SNRK protein is expressed in E17.5 mouse embryos in the aortic endothelial and smooth muscle cells, in the epithelial cells of the esophagus and lung, and CMs as well as the vascular cells (endothelial cells and smooth muscle cells) of the heart. Expression data support the argument that *Snrk* is expressed at both mRNA and protein levels in tissues that have a high demand for metabolic activity during embryonic development. Therefore, it is not surprising that global loss of *Snrk* leads to neonatal lethality some time after birth and P1. The next question is what is the cause of this lethality? The *Snrk* KO heart size, which is significantly larger than *Snrk* HET or WT mice, provides some clues. Increased heart size could be the direct result of increased proliferation or decreased apoptosis of CMs, which was not observed. Investigation of *Snrk* KO E17.5 and P0 heart display decreased glycogen storage. Interestingly, E17.5 *Snrk* KO hearts do not show lipid staining defects but P0 hearts do. In addition to heart, P0 *Snrk* KO liver and intestine also shows decreased lipid staining. Supporting the lipid and glycogen storage defects in *Snrk* KO heart is the microarray data that indicate alterations in lipid, and carbohydrate metabolic gene pathways. Further, decreased circulating triglycerides and free fatty acids in *Snrk* KO neonate blood bolsters this notion. These results imply that the heart in response to decreased availability of circulating lipids for FAO, is unable to activate compensatory stress mechanism to increase efficient energy production resulting in eventual neonatal lethality. This poor metabolic ability of the developing heart led to an embryonic/fetal cardiomyopathy. Human fetal cardiomyopathies are poorly understood and metabolic abnormalities are not typical; however, there have been case reports that have documented metabolic abnormalities as a cause of fetal cardiomyopathy (de Koning et al., 1998; Patel et al., 1999; Steenhout et al., 1990). It is also noteworthy that *Snrk* KO pups do contain observable milk spots which suggest they are they able to feed, and therefore neonatal death is not due to insufficient nourishment from mother.

Because of the global nature of the *Snrk* KO mice, it is difficult to assess the contribution of *Snrk* from other tissues to this metabolic phenotype. To assess this, we conditionally deleted *SNRK* in CMs in vitro, as well as in vivo using MYH6CRE (Agah et al., 1997) and TIE2CRE (Kisanuki et al., 2001) mouse lines respectively. We observed metabolic abnormalities in in vitro cultured CMs and cardiac tissue isolated from TIE2CRE mouse line. Additionally, the conditional loss of *Snrk* in CMs in vivo resulted in severe cardiac functional defects that resulted in adult lethality reinforcing the importance of SNRK in CMs. Both heterozygous global KO (data not shown) and homozygous conditionally deleted adult *Snrk* mice show cardiac function deficits without any applied stress arguing that *Snrk* is a highly regulated gene during cardiac metabolism. Collectively, our data suggest a strong correlation to cardiac metabolism alteration as one reason for the *Snrk* KO mice neonatal lethality.

What is the possible mechanism that explains the cardiac metabolism defects? During embryonic development, the cardiac tissue uses glucose as the primary source of energy, which switches to free fatty acids in the neonate and adult (Breckenridge et al., 2013; Lopaschuk and Jaswal, 2010). This switch in metabolic source has profound impact in downstream signaling cascades in cells such as CMs that are responsible for the major metabolic function of the heart. In neonates, the fatty acids are transported into the cytoplasm via fatty acid transporter, and are subsequently converted to acyl-CoA. Acyl-CoA is transported into the mitochondria for β-oxidation via carnithine palmitoyl transferase (CPT). FAO/β-oxidation converts acyl-CoA into acetyl-CoA, which can enter the tricarboxylic acid (TCA) cycle for ATP generation. One of the rate limiting steps in β-oxidation is the inhibition of CPT by Malonyl-CoA. Malonyl-CoA is generated from acetyl-CoA by an enzyme called acetyl-CoA carboxylase (ACC) (Folmes and Lopaschuk, 2007). Phosphorylation of ACC is inhibitory in that it blocks Malonyl-CoA synthesis, which eliminates CPT inhibition and increases FAO and thus leading to increased acetyl-CoA and ATP generation via TCA cycle. In E17.5 *Snrk* KO hearts we noticed a decreasing trend in pACC, which was statistically significant in P0 neonates. pAMPKα a regulator of pACC (Folmes and Lopaschuk, 2007) was also lower at E17.5 in *Snrk* KO hearts. Conditional deletion of *Snrk* showed altered pACC and pAMPKα levels in endothelial specific but not CM-specific neonatal mouse hearts. Interestingly, the *SNRK* knockdown CMs showed similar defects with decreased FAO as the global and endothelial conditional KO mouse (Fig. 8). Recently, endothelial cells have been shown to be glycolytic (De Bock et al., 2013), but the functional consequences of disrupting metabolism in endothelial cells are not known. By knocking out *Snrk* specifically in endothelial cells, we demonstrate a cardiac defect namely in activation of AMPK and fatty acid metabolism, resulting in decreased energetics and endothelial metabolism.

At a cardiac functional level, it is noteworthy that the *Snrk* CM-specific KO develops cardiomyopathy (Fig. 9), and assuming that this condition is metabolism-induced, we hypothesize that the CM-specific SNRK contribution will unravel in mice once they are past the neonatal stage, a development time when cardiomyopathic hearts usually switch metabolic preference from FAO back to glycolysis as a principal energy source (Akki et al., 2008; Allard et al., 1994; Kalsi et al., 1999; Kolwicz et al., 2013). We therefore hypothesize that endothelial SNRK may be masking CMs SNRK-specific signaling pathway effects in vivo. This hypothesis is partly supported by *SNRK* knockdown CMs results in vitro where endothelial cells are not included in the experimental design. Other possibilities include SNRK participate in alternative signaling pathways such as PPARα that is known to play a role in FAO in CMs and ECs (Belanger et al., 2002; Schrammel et al., 2014).

To unravel the mechanistic details of how SNRK interferes with pACC-pAMPK pathway in CMs, we performed chemical activator experiments with Metformin (indirect AMPK activator) and AICAR (direct AMPK activator) treatment in control or *SNRK* shRNA knockdown human embryonic stem cell differentiated CMs (Fig. 7). Interestingly, Metformin and AICAR showed different results in that Metformin rescues pACC and pAMPKα levels, while AICAR does not rescue pACC levels in CMs. Metformin can activate AMPK by different mechanisms, it can inhibit complex 1 of the mitochondrial respiratory chain causing in an increase in AMP levels which results in the activation of AMPK (El-Mir et al., 2000; Hawley et al., 2010; Owen et al., 2000) or Metformin can activate LKB1 (Garrido-Urbani et al., 2011; Rattan et al., 2011; Xie et al., 2008), a tumor suppressor kinase that is mutated in Peutz-Jeghers Syndrome (Hemminki et al., 1998), and an upstream regulator of SNRK and AMPK. Because LKB1 is known to phosphorylate SNRK directly on threonine residue 173 in HeLa cells (Jaleel et al., 2005), as well as AMPKα on threonine residue 172 (Sanders et al., 2007), the rescue for AMPK in metformin treated CMs suggests that SNRK is necessary but not required for this activation. Interestingly, the finding that AICAR does not rescue pACC phenotype suggests that SNRK is necessary for ACC inhibition in CMs, independent of AMPK. The AICAR and Metformin data together imply that SNRK participates in the AMPK-ACC signaling axes either by directly phosphorylating AMPK whereby triggering phosphorylation of ACC or by directly phosphorylating ACC (supplementary material Fig. S8). Collectively, our data argue that in *Snrk* KO neonates, the heart is undergoing stress due to decreased capacity for FAO, and decreased available circulating fatty acids for carrying out FAO. Targeting the LKB1-AMPK pathway has shown to inhibit tumor growth (Nagalingam et al., 2012), and the findings here provide rationale for SNRK as a target at the interface of metabolism and angiogenesis associated with tumor growth.

In summary, this study reports a highly expressed AMPK family member *Snrk* in mammals that is responsible for cardiac metabolic homeostasis, and deficiency in *Snrk* results in cardiac insufficiency resulting from deficits in FAO. Future work will focus on the genes and pathways that cross talk with SNRK to contribute to this defect.

## Supplementary Material

Supplementary Material
