# Supplementary material for: Sucrose non-fermenting related kinase enzyme is essential for cardiac metabolism
Source: Biol Open. 2014 Dec 12;4(1):48–61. doi: 10.1242/bio.20149811 (PMC4295165; doi:10.1242/bio.20149811)
Supplement: Supplementary Material [file supp_4_1_48__index.html]

Sucrose non-fermenting related kinase enzyme is essential for cardiac metabolism — Supplementary Material 

# *Sucrose non-fermenting related kinase* enzyme is essential for cardiac metabolism

## bio.20149811 Supplementary Material

**Files in this Data Supplement:**

- Supplementary Material - Stephanie M. Cossette et al. doi: 10.1242/bio.20149811
- Table S2 - **List of metabolic genes identified in microarray analysis of *Snrk* E17.5 mouse hearts.**
