# Supplementary material for: Sucrose non-fermenting related kinase enzyme is essential for cardiac metabolism
Source: Biol Open. 2014 Dec 12;4(1):48–61. doi: 10.1242/bio.20149811 (PMC4295165; doi:10.1242/bio.20149811)
Supplement: Supplementary Material [file supp_bio.20149811_bio.20149811-s1.pdf]

Biology Open (2015) 000, 1–14 doi:10.1242/bio.20149811

Supplementary Material

Stephanie M. Cossette et al. doi: 10.1242/bio.20149811

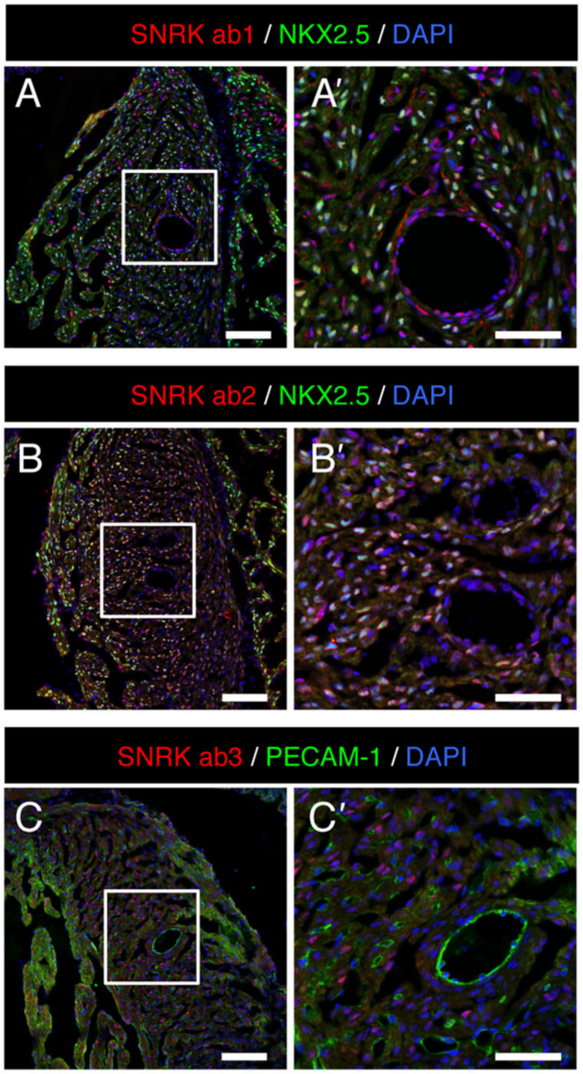

Fig. S1. Immunostaining for SNRK using different commercially available SNRK antibodies in neonate heart tissue sections. (A–C) Immunofluorescence analysis of transverse sectioned wild-type neonate hearts stained with the SNRK antibody (red) the cardiomyocyte marker NKX2.5 (green), the endothelial cell marker PECAM-1 (green) and the nuclear DNA marker DAPI (blue). (A′–C′) High power magnified images of boxed regions in panels A–C. ab1 SNRK antibody Abcam ab96762; ab2 SNRK antibody Abcam ab94540; ab3 SNRK antibody Abgent AP7249c. Scale bars: 100 µm.

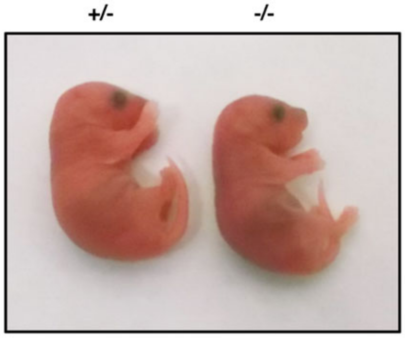

Fig. S2. *Snrk* KO live pups do not display obvious gross morphological defects. Photographs of live neonates *Snrk* HET (+/−) and KO (−/−).

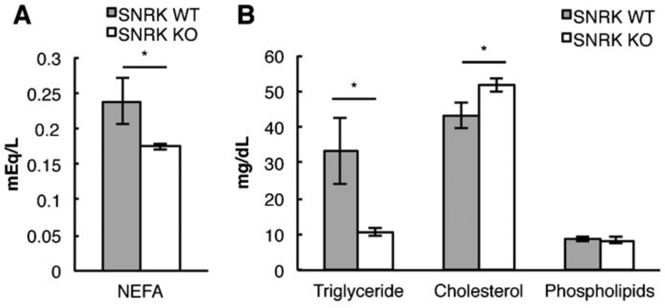

Fig. S3. Loss of *Snrk* results in circulating lipid defects. Blood plasma was isolated from *Snrk* WT n=6 and *Snrk* KO n=6 neonates and was assayed for NEFA (mEq/L), triglyceride (mg/dL), phospholipids (mg/dL) and cholesterol (mg/dL). There was a significant decrease in the mean levels of both NEFA (A) and Triglycerides (B) in *Snrk* KO neonates (WT NEFA 0.239 mEq/L, TGs 33.33 mg/dL; KO NEFA 0.175 mEq/L, p=0.040, TGs 10.63 mg/dL, p=0.018). There was a slight increase in the mean levels in cholesterol (WT 43.45 mg/dL; KO 51.58 mg/dL, p=0.038) (B) in neonates and no difference in the mean levels of phospholipids in *Snrk* WT and KO neonates (WT 8.80±0.81 mg/dL; KO 8.37±0.82 mg/dL; p=0.360). * *p-value*<0.05.

Biology Open (2015) 000, 1–14 doi:10.1242/bio.20149811

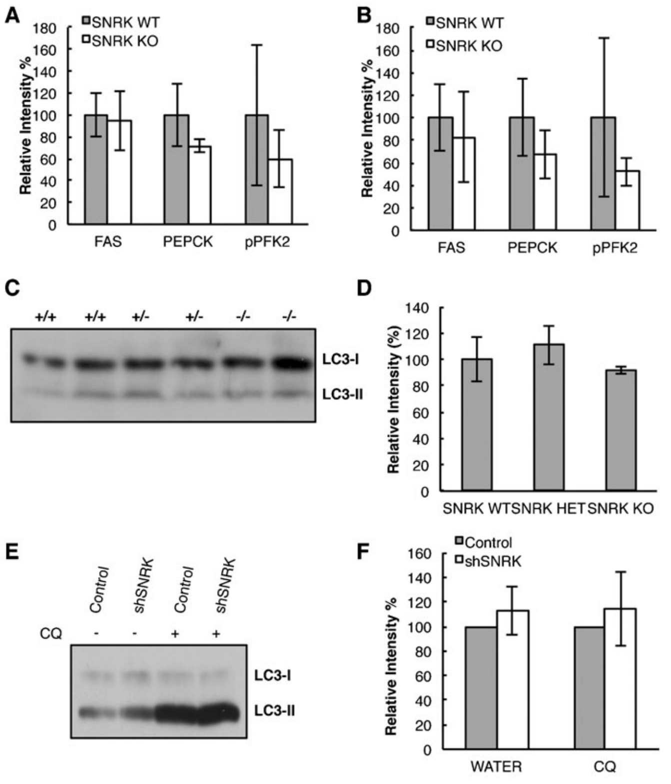

Fig. S4. Loss of *Snrk* does not result in changes in glycolysis, gluconeogenesis, fatty acid synthesis or autophagy. The expression of FAS, PEPCK, pPFK2 in E17.5 (A) and neonate (B) heart lysates from *Snrk* WT and KO hearts was determined by western blotting followed by densitometry quantitation using tubulin as the loading control. The results are the mean ± SEM from three independent embryonic hearts for each genotype. FAS and PEPCK quantification was normalized to tubulin, pPFK2 was normalized to PFK2. (C,D) The expression of LC3-I and LC3-II in lysates from *Snrk* WT (+/+), HET (+/−) and KO (−/−) neonatal hearts was determined by western blotting (C) followed by densitometry quantitation (D). The results are the mean ± SEM of the LC3-II/LC3-I ratio from three independent embryonic hearts for each genotype. (E) The expression of LC3-I and LC3-II in hESC-derived CMs infected with empty vector shRNA control (Control) lentivirus and *SNRK* shRNA lentivirus (shSNRK) was determined by western blotting followed by densitometry quantitation (F). The CMs were serum starved for 4 hours to induce autophagy and treated with either Water or 25 µM Choloroquine (CQ). The results are the mean ± SEM of the LC3-II/LC3-I ratio from three independent cardiomyocyte differentiations and infections.

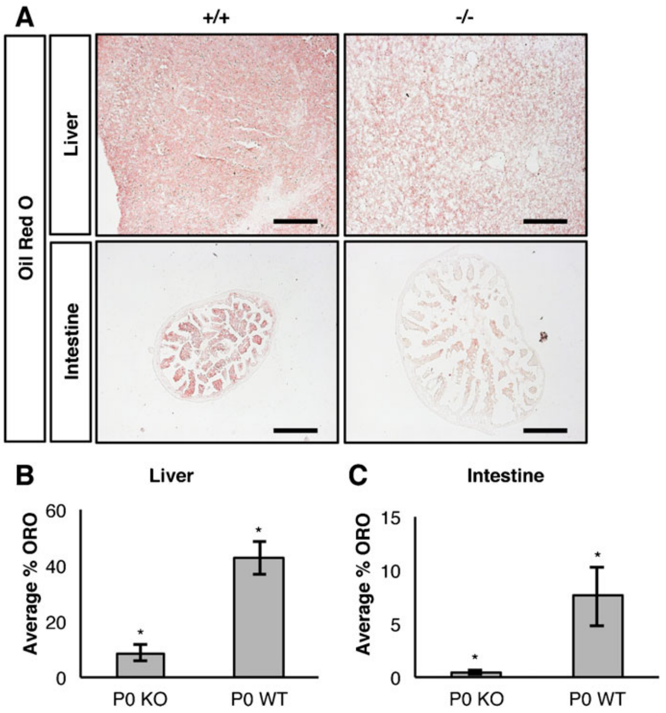

Fig. S5. Loss of *Snrk* results in lipid accumulation defects in neonate liver and intestine. (A) Photomicrograph of sectioned liver and intestine isolated from *Snrk* WT and *Snrk* KO neonate mice. Sections subjected to Oil Red O staining. Quantification of the percentage of ORO staining in liver (B) and intestine (C) was conducted by calculating the amount of red ORO stain divided by the total area of the liver or intestine. Intestine WT 7.57%; KO 0.42%, $p=0.04$, n=3, and Liver WT 43%; KO 9%, $p=0.0021$, n=3. Scale bars: 250 µm. * $p\text{-value}<0.05$.

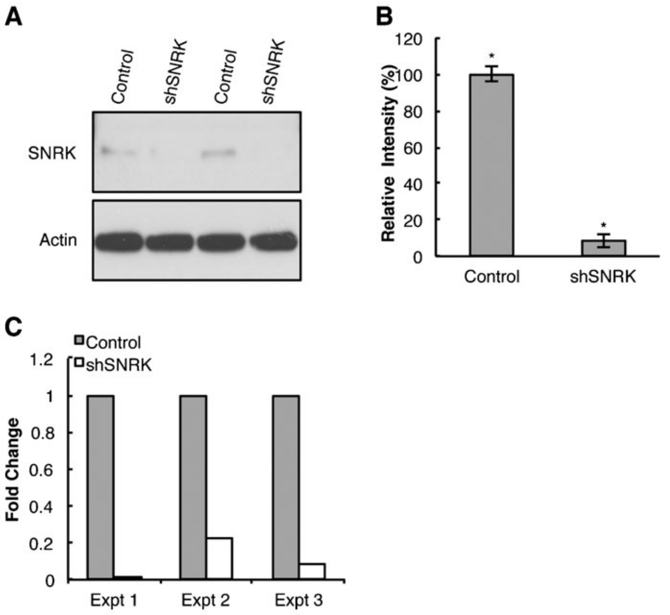

Fig. S6. shRNA SNRK knockdown validation in HUVECs and hESC derived cardiomyocytes. The expression of SNRK in human umbilical vein endothelial cells (HUVECs) infected with empty vector shRNA control (Control) lentivirus and *SNRK* shRNA lentivirus (shSNRK) was determined by western blotting (A) followed by densitometry quantitation using actin as the loading control (B). The results are the mean ± SEM. * *p-value*$<$0.05. (C) The fold change in the mRNA expression level of *SNRK* in hESC derived cardiomyocytes was determined using qPCR analysis from cells infected with Control or shSNRK in three independent cardiomyocyte differentiations.

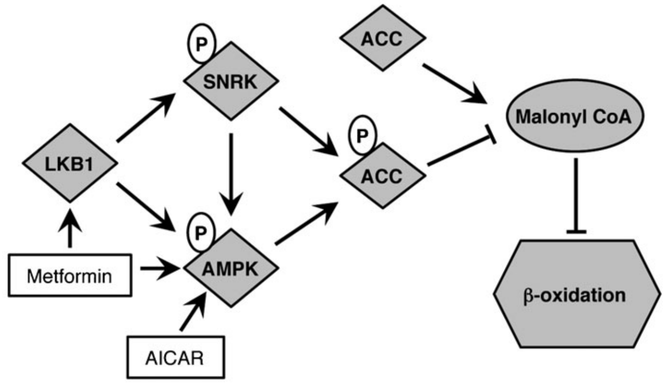

Fig. S8. Proposed model for the mechanism of SNRK FAO regulation. Both SNRK and AMPKα are phosphorylated and activated by LKB1. Once phosphorylated, SNRK is able to directly or indirectly regulate the phosphorylation of AMPKα. Phosphorylated AMPKα is can directly phosphorylate ACC resulting in an inhibition of Malonyl CoA production and an increase in β oxidation. SNRK independent of phosphorylated AMPKα is also able to regulate the phosphorylation levels of ACC.

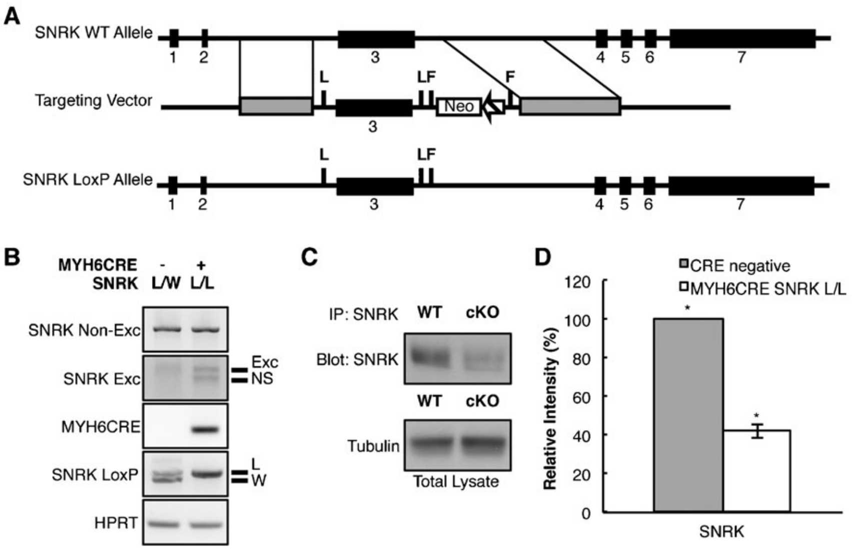

Fig. S7. Conditional *Snrk* LoxP gene targeting and confirmation. (A) Schematic illustration of the strategy used to generate the Conditional *Snrk* LoxP mice. *Snrk* exon 3 was targeted and replaced with *Snrk* exon 3 flanked by LoxP sites as well as a neomycin cassette flanked by FRT sites. The neomycin (Neo) cassette was removed by FRT genomic recombination generating the *Snrk* LoxP allele. (B) PCR based genotyping indicates excision of *Snrk* Exon 3 in DNA isolated from MYH6CRE *Snrk* LoxP/LoxP neonates. The SNRK non-excised (Non-Exc) band is present in both CRE− and CRE + hearts which indicates that there is an unexcised locus in both DNA samples. The SNRK excised (Exc) band is only present in the CRE + DNA sample indicating that successful genomic recombination has occurred in the MYH6CRE positive cardiomyocytes. (C,D) SNRK protein expression is decrease in MYH6CRE positive SNRK LoxP/LoxP (cKO) adult heart lysates compared to CRE negative wildtype (WT) adult hearts. (C) SNRK immunoprecipation using anti-SNRK antibody followed by western blot analysis with anti-SNRK. Total lysates were immunoblotted for Tubulin. (D) Densitometry quantitation of SNRK expression normalized to the densitometry quantitation of Tubulin in the total lysates. The results are the mean ± SEM from three independent adult heart lysates for each genotype. * *p-value*$<$0.05. F, FRT; NS, non-specific band; L, LoxP band; W, WT band.

Biology Open (2015) 000, 1–14 doi:10.1242/bio.20149811

Table S1. Loss of *Snrk* results in neonatal lethality during postnatal day 0

		Genotype		
Mouse stage	Number genotyped	+/+	+/−	−/−
E10.5	24	45.83% (11)	33.33% (8)	20.83% (5)
E12.5	64	25.00% (16)	46.88% (30)	28.13% (18)
E15.5	36	22.22% (8)	55.56% (20)	22.22% (8)
E17.5	230	23.48% (54)	57.39% (132)	19.13% (44)
P0	138	24.64% (34)	56.52% (78)	18.84% (26)
P1	36	38.89% (14)	55.56% (20)	5.56% (2)
P2	20	35.00% (7)	65.00% (13)	0.00% (0)
P21	56	42.86% (24)	57.14% (32)	0.00% (0)

Table S2. List of metabolic genes identified in microarray analysis of *Snrk* E17.5 mouse hearts. See supplementary webpage.

Table S3. Loss of *Snrk* does not result in neonatal lethality in cardiac specific or endothelial specific loss of *Snrk*

		Genotype		
Mouse line	Age	CRE neg	CRE L/WT	CRE L/L
MYH6CRE	P0	49% (47)	29% (28)	22% (21)
	P21	46% (44)	29% (28)	25% (24)
TIE2CRE	P0	64% (25)	23% (9)	13% (5)
	P21	48% (22)	39% (18)	13% (6)
Expected		50%	25%	25%
